# Human pluripotent stem cell-derived retinal ganglion cells: advances in differentiation and translational applications

**DOI:** 10.1186/s10020-025-01405-0

**Published:** 2025-12-08

**Authors:** Jessica Yuen Wuen Ma, Maciej Daniszewski, Alice Pébay

**Affiliations:** 1 Department of Anatomy and Physiology, The University of Melbourne, Melbourne, VIC Australia; 2 Department of Surgery, Royal Melbourne Hospital, The University of Melbourne, Melbourne, VIC Australia; 3 CellTellus Laboratory, Melbourne, VIC Australia

**Keywords:** Retinal ganglion cells, Human pluripotent stem cells, Retinal organoids, Transplantation

## Abstract

Retinal ganglion cells (RGCs) are neurons that transmit visual information from the retina to the brain. Their degeneration, as seen in glaucoma and other optic neuropathies, leads to irreversible vision loss. As mature human RGCs are difficult to access, most of their studies rely on rodent models, which do not fully recapitulate human retinal biology. Human pluripotent stem cells (hPSCs) provide a promising source for generating RGCs in vitro, supporting disease modelling, drug screening, and future cell replacement therapies. This review outlines key markers that define RGC identity, maturation stages, and subtype diversity. We summarise recent advances in the differentiation of hPSCs towards RGCs, their functional characterisation, and their applications in disease modelling, drug screening, and transplantation.

## Introduction

Retinal ganglion cells (RGCs) are neurons located in the innermost layer of the retina. They are essential for vision, transmitting visual information from photoreceptors to the brain via the optic nerve [1]. The human retina contains approximately 1.2 million RGCs split into subtypes with distinct functions. Previous studies identified 40–46 RGC subtypes in the mouse retina [2, 3] and 18 subtypes in primates [4]. However, the exact number of subtypes in the human retina is currently unknown. In the human retina, RGCs collectively preprocess and relay signals from 120 million rod photoreceptors and 6 million cone photoreceptors [1]. This encoding supports key visual functions such as spatial acuity, motion detection, and colour vision, with different RGC subtypes tuned to transmit distinct visual features (e.g., form, colour, motion) to the brain [1]. Given these roles, damage or loss of specific RGC subtypes has serious consequences, as degeneration of their axons or cell bodies severs communication with the brain, leading to visual field deficits and potentially irreversible blindness [5].

RGCs are particularly vulnerable to injury and neurodegenerative diseases due to their postmitotic nature and long axons [6]. Glaucoma is the most prevalent disease of the optic nerve, often associated with elevated intraocular pressure (IOP), and remains the leading cause of irreversible blindness globally [7]. The number of people aged 40–80 affected by glaucoma was approximately 76 million in 2020, with estimates exceeding 111 million by 2040 due to population ageing [8]. Treatments lowering IOP can slow progression, yet they do not halt RGC loss, as these neurons cannot regenerate once damaged [9]. Understanding RGC degeneration and developing preventative and regenerative strategies are therefore urgent research priorities. Much of our knowledge of RGC biology comes from rodent models, which have revealed pathogenic pathways and potential regenerative targets [10–12]. However, significant differences between rodent and human RGCs, including limited cross-species conservation of RGC subtypes, reduce the translational relevance of rodent models [4, 13–16]. This underscores the need for human-specific models.

Human pluripotent stem cells (hPSCs), including human embryonic stem cells (hESCs) and human induced pluripotent stem cells (hiPSCs), offer a promising alternative. hiPSCs can be generated from adult somatic cells through reprogramming with defined transcription factors [17, 18]. They differentiate into cells of all three germ layers, enabling the generation of RGCs in a patient-specific manner. This makes them a powerful tool for patient-specific disease modelling, drug screening and potential cell replacement therapies. Nevertheless, before any therapy is developed, it is essential to generate RGCs that express the correct molecular markers and demonstrate functional properties.

Here, we summarise recent advances (2020–2025) in RGC differentiation from hPSCs, including their molecular characterisation, and the comparative advantages and limitations of different in vitro culture systems. We also present information on enrichment methodologies to increase RGC yield, alongside with functional assays for RGC identity validation. We further review insights from comparative transcriptomics, functional assays, and subtype profiling, and discuss the translational applications of hPSC-derived RGCs in disease modelling, drug screening, and transplantation.

## Markers for RGC differentiation

**Fig. 1:**
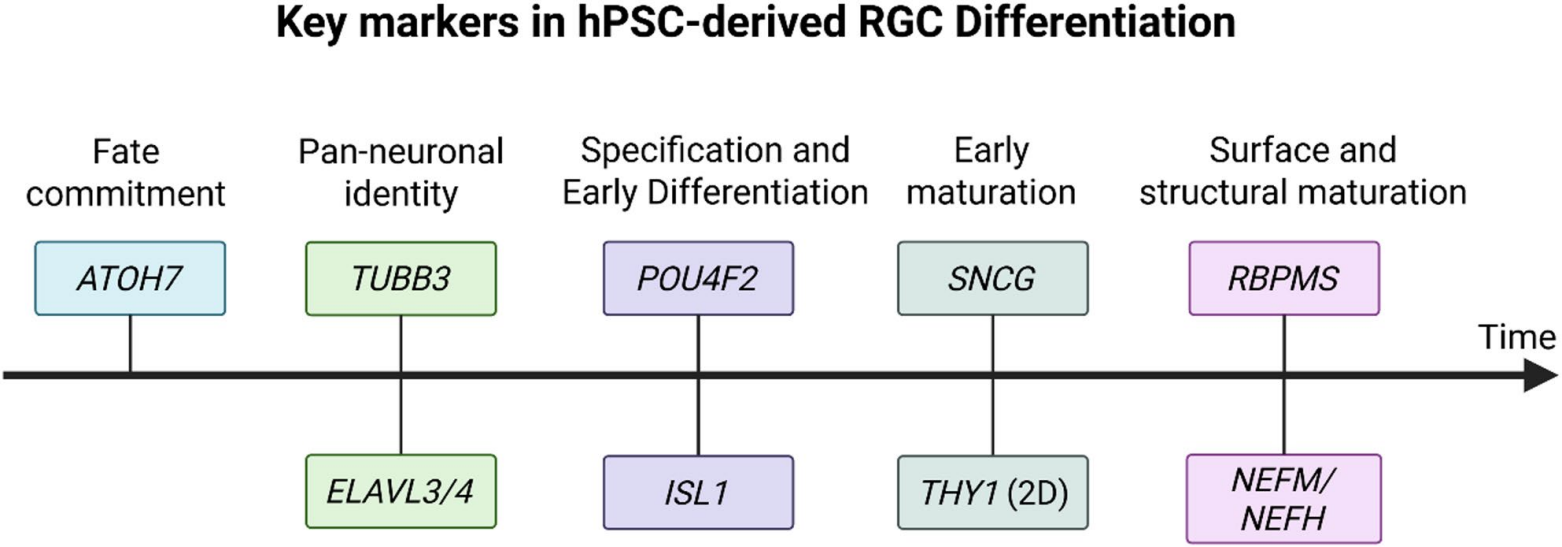
Schematic timeline illustrating the sequential expression of key RGC markers during hPSC-RGC differentiation. The early RGC competence factor *ATOH7* is expressed first in retinal progenitors, followed by general neuronal markers *TUBB3* and *ELAVL3/4* as cells exit the cell cycle and commit to a neuronal lineage. RGC specification factors *POU4F2* and *ISL1* appear next, marking the onset of RGC identity. Maturation markers such as *SNCG* and *THY1* emerge as RGCs begin neurite outgrowth, followed by late-stage markers *RBPMS*, *NEFM*, and *NEFH*, which indicate structural and axonal maturation. Figure created with BioRender.com

The identification of hPSC-derived RGCs relies on the confirmed expression of specific molecular markers. However, many markers are not unique to RGCs and may also be expressed by other neuronal or sensory cell types outside the retina, such as POU Class 4 Homeobox 1 (POU4F1/BRN3A) in auditory neurons [19] and ISL LIM homeobox 1 (ISL1) in motor neurons [20]. Therefore, it is critical to first confirm that hPSCs have committed to a retinal lineage. In three-dimensional (3D) systems such as retinal organoids, this specification typically occurs as part of the self-organising retinogenesis process [21]. In contrast, two-dimensional (2D) differentiation systems require confirmation of retinal identity, often through the detection of eye field markers [22]. Since individual RGC markers often lack specificity, identifying RGCs depends on the co-expression of multiple markers, particularly when retinal differentiation is incomplete or inefficient. The temporal sequence of marker expression during hPSC-derived RGC specification is summarised in Fig. 1.

### Fate commitment and RGC specification

Atonal BHLH Transcription Factor 7 (ATOH7) is a transcription factor expressed transiently in retinal progenitor cells as they begin to adopt an RGC fate [23, 24]. The POU Class 4 homeobox (POU4/BRN3) transcription factor family, which includes POU4F1 and POU Class 4 Homeobox 2 (POU4F2/BRN3B), and POU Class 4 Homeobox 3 (POU4F3/BRN3C), is sequentially expressed during RGC development, with POU4F2 appearing first and POU4F1 one day later [25]. Both POU4F1 and POU4F2 are critical for RGC specification and maturation [24, 26] and are considered general RGC markers, whereas POU4F3 is restricted to a smaller RGC subset and its deletion in mice has little effect on RGC survival [27, 28]. ISL1 is a transcription factor commonly co-expressed with POU4F2 in early differentiating RGCs, but also labels other inner retinal neurons, including bipolar, interneural, and amacrine cells [29–31]. In hPSC cultures, POU4F2 and ISL1 are reliably induced within the first 4–6 weeks, but cell numbers often decline in long-term organoid culture [24, 32].

### Pan-neuronal differentiation and early maturation

General neuronal proteins, such as βIII-Tubulin (*TUBB3*) and HuC/D (*ELAVL3/4*), confirm neuronal differentiation but lack specificity [33, 34]. As a structural protein, βIII-Tubulin is expressed in nearly all immature neurons of the central nervous system [35], and certain amacrine cells, horizontal cell processes, and cone photoreceptors [36]. Similarly, the HuC/D protein is found in ganglion cells, amacrine cells, and transiently in horizontal cells [37]. In contrast, γ-Synuclein (SNCG) is highly enriched in RGCs and, when combined with POU4F1, provides strong evidence of RGC identity [38, 39]. Thy-1 cell surface antigen (THY1/CD90) is a glycophosphatidylinositol-linked surface protein historically known as an RGC-specific antigen in retina [40]. It is widely used for RGC isolation in 2D systems [41, 42]. This approach is also effective for RGCs obtained from dissociated retinal organoids, which begin to express surface THY1 within days of being re-plated under 2D conditions [43]. However, THY1 protein expression is often absent or very low in 3D organoids [43–45]. This suggests that the 3D environment or developmental stage may not support THY1 expression. Therefore, while THY1 is effective for isolating RGCs in 2D systems, its absence in 3D systems should not be interpreted as a lack of RGCs.

### RGC maturation and structural identity

RNA-binding protein with multiple splicing (RBPMS) is a well-established pan-RGC marker, selectively expressed in nearly all RGCs across multiple mammalian species [46]. In 2D hPSC-derived RGC cultures, RBPMS levels are often low. By day 35, only 22% of hiPSC-derived RGCs expressed RBPMS, despite over 85% expressing POU4F, identified with a pan-POU4F antibody or other RGC markers [47]. Similarly, in day 80 cultures from dissociated day 40 retinal organoids, *RBPMS* expression was lower than *POU4F2*, *ISL1*, and *SNCG* [48], suggesting RBPMS may underestimate RGC numbers in vitro. In retinal organoids, RBPMS is barely detectable during the early weeks of development [44]. POU4F^+^ RGCs appear two weeks before RBPMS expression becomes abundant [44]. This delayed and inconsistent expression pattern underscores the limitations of RBPMS as a sole marker for identifying hPSC-derived RGCs in vitro. Neurofilament proteins such as neurofilament medium chain (*NEFM*) and neurofilament heavy chain (*NEFH*) confirm RGC axonal outgrowth and maturation [49]. However, neurofilament proteins are not specific to RGCs, as they are expressed by other projection neurons with long processes. In retinal tissue, neurofilament markers label all axons in the nerve fibre layer, including but not limited to RGCs [50].

### Summary of marker expression differences between culture systems (Table 1)

**Table 1:** Summary of key RGC markers and their expression in 2D vs. 3D hPSC-derived systems

Marker	Function/Notes	Expression in 2D Cultures	Expression in Organoids
ATOH7	Early RGC lineage marker; precedes POU4F	Early retinal progenitor cells marker	
POU4F2	RGC-specific transcription factor; drive early differentiation	Present by 4–6 weeks	Peaks around day 35; then declines
ISL1	Early RGC marker; also in other retinal neurons	Co-expressed with POU4F2	Detected with POU4F2 early
βIII-Tubulin	Early neuronal marker; not RGC-specific	Expressed in neurons	
HuC/D	Postmitotic neuron marker; not RGC-specific	Expressed in neurons	
SNCG	Highly specific to RGCs	Co-expressed with POU4F1	Detected in POU4F1^+^ cells at inner surface
THY1 (CD90)	Surface protein of RGCs	Present	Absent or very low; minimal mRNA and undetectable protein
RBPMS	Mature RGC marker	Often low	Low early; increases after 6 weeks
NEFM/NEFH	Cytoskeletal markers; not RGC-specific	Upregulated as RGCs extend neurites	Expressed in mature axon-forming neurons

RGCs can be generated from monolayer or organoid cultures, but their marker expression profiles often differ. In 2D cultures, directed differentiation using small molecules lead to rapid induction of POU4F2 expression within 4 to 6 weeks [47]. Surface markers like THY1 are readily induced and commonly used for RGC isolation [41, 42]. RBPMS is often lower or delayed in these cultures. In contrast, 3D system better recapitulate in vivo retinogenesis, showing a stepwise emergence of RGC markers: ATOH7 in retinal progenitors, followed by POU4F and ISL1, and later RBPMS and neurofilament proteins as the cells mature [44]. THY1 is typically low or undetectable in retinal organoids but can be enriched after dissociation and replating in 2D [43].

## Current approaches for differentiating RGCs from hPSCs (Fig. 2)

**Fig. 2:**
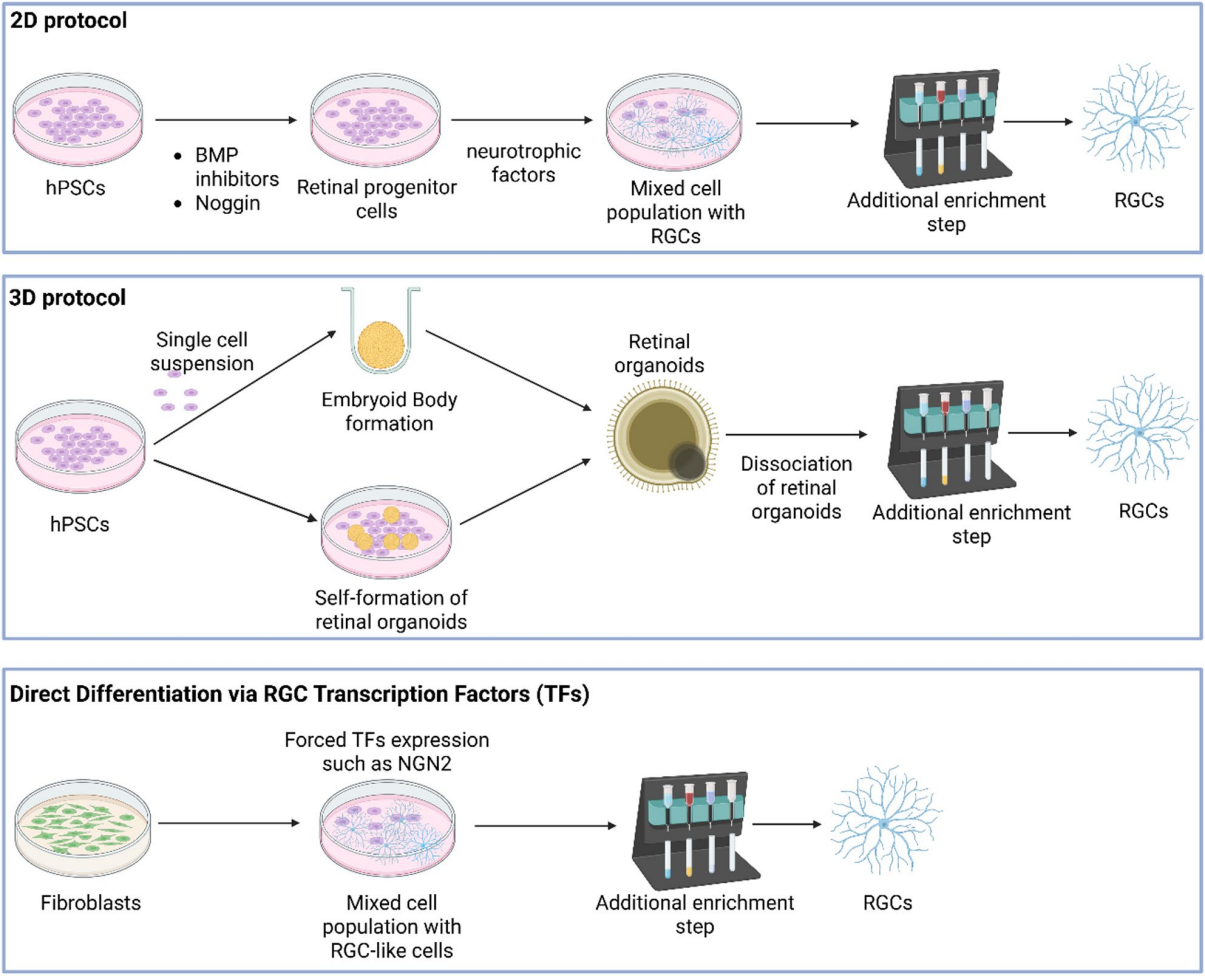
Schematic summary of the 2D protocols, 3D protocols and direct differentiation using RGC transcription factors used to generate RGCs in vitro. Figure created with BioRender.com

We have focused on studies published within the past five years (2020–2025). For protocols developed prior to this period, readers are referred to previously published review articles [8, 51–55].

### Adherent differentiation protocols

2D protocols culture guide hPSCs through a monolayer differentiation process that mimics in vivo retinal development by applying staged growth factor and small-molecule treatments [42]. Differentiation typically begins with neuroectoderm induction, achieved via dual SMAD inhibition to block BMP and TGF-β/Activin signalling. This is commonly achieved using Noggin or LDN-193,189 (BMP inhibitors) together with SB431542 or A83-01 (TGF-β/Activin inhibitors) [56–58]. Concurrent suppression of Wnt signalling, often through DKK1 or XAV939, promotes anterior neural identity [59]. One example of a purely 2D differentiation protocol is that described by Chavali et al. [42]. In this approach, hiPSCs are grown to confluence, and from day 0 to 4, treated with a cocktail of inhibitors and supportive factors, including LDN-193,189, SB431542, XAV939, together with brain-derived neurotrophic factor (BDNF) and ciliary neurotrophic factor (CNTF) [42]. From day 4 to 21, nicotinamide is removed and bFGF added to promote the expansion of retinal progenitor cells. This approach results in the expression of early retinal progenitor cell markers in over 95% of cells by day 7. Subsequently, from day 22 to 36, differentiation is directed towards the RGC lineage using neurotrophic factors such as BDNF and CNTF, and Shh or Notch pathway inhibitors such as DAPT [42]. This differentiation method yields RGCs with 40–50% THY1^+^, 82–84% POU4F2⁺ and 11–12% RBPMS^+^ RGCs by day 35 [42]. Several other 2D protocols are based on this framework [32, 47, 60]. One such improvement reports a streamlined 2D protocol in which over 80% of cells expressed PAX6⁺ by day 9 and gave rise to RGCs within four weeks [32].

In addition to direct differentiation from hiPSCs, a recent study by Gozlan et al. (2023) described a 2D adherent protocol that enables the generation of RGCs from banked hiPSC-derived retinal progenitor cells. These retinal progenitor cells maintained their multipotent phenotype after cryopreservation and, upon thawing, could be directly differentiated under adherent conditions into multiple retinal lineages, including RGCs. When cultured in basal medium, the cells upregulated RGC markers, with approximately 8% BRN3A⁺ cells among PAX6⁺ progenitors within one week, demonstrating the feasibility of generating RGCs from previously cryopreserved retinal progenitor cell stocks. This approach offers a time-efficient alternative for obtaining functional RGCs within a few weeks, bypassing the need for differentiation directly from hiPSCs [61].

### Retinal organoids and suspension cultures

3D protocols generate self-organising retinal organoids from hPSCs in suspension culture, recapitulating the layered architecture of the retina [62, 63]. These protocols typically begin with the aggregation of hPSCs into embryoid bodies and differentiated into retinal organoids using early signalling steps similar to those in 2D systems, i.e. neural induction via dual SMAD inhibition and eye-field specification using factors such as IGF-1, BMP4, or Wnt inhibitors [64, 65]. Over several weeks, these organoids develop stratified structures resembling the embryonic retina, with proliferative retinal progenitors and differentiating neurons arranged in distinct zones [66]. Notably, RGCs are the first neuronal subtype to emerge, forming in the innermost layer [67–69]. Although RGCs form robustly at this stage, even with organoids were replated onto Matrigel-coated dishes to promote neurite extension and enrichment, they still constituted only 17% of total cells in 49-day-old organoids, as confirmed by single-cell RNA sequencing (scRNA-seq) [70].

Protocols relying on the innate self-patterning capacity of hPSC aggregates with minimal added factors have also been reported. Wagstaff et al. [24] reported successful RGC generation within four weeks using a modified basal medium and controlled aggregation, without the use of these inhibitors. By week 4, over 30% of the cells were POU4F2⁺/SNCG⁺ and over 50% were ATOH7⁺, as determined by immunocytochemistry [24]. At later stages of differentiation, organoids are typically maintained in rotating or static suspension culture and transitioned to retinal differentiation media containing supplements such as B27, taurine, and retinoids [24].

#### Limitations of retinal organoids

Due to the directed nature of the differentiation, retinal organoids typically lack cells of non-neuroectodermal origin. The absence of vasculature significantly restricts nutrient diffusion to the innermost regions of the retinal organoid, leading to core necrosis and loss of inner retinal layers during long-term culture [71]. Moreover, growth factors derived from endothelial cells have been shown to promote self-renewal and neurogenesis of neural stem cells [72] which may have a notable impact on the differentiation within the organoid and will prevent the core necrosis. Indeed, vascularisation of retinal organoids has recently been shown to increase their size and maturation, as exemplified by the increased expression of SNCG and SMI-32 in vascularised organoids compared to non-vascularised ones [73].

Another limitation is the absence of microglia, which regulate neuronal survival and synapse formation [74, 75], and play a role in patterning the vasculature through programmed cell death [76]. Recent studies have reported successful integration of microglia into retinal organoids, with microglial migration to the outer plexiform layer, and their ability to phagocytose RGCs and respond to an induced immune challenge with LPS, TLR3-activating compound POLY(I: C) or *E. coli* [77–79].

Nevertheless, adding vasculature and microglia into retinal organoids may not be enough to address another limitation of retinal organoids that is the progressive degeneration of RGCs, especially in older organoids [80, 81]. A potential cause of this phenomenon may be the lack of signals from RGC post-synaptic targets. It is known that during development RGCs receive survival signals from their primary post-synaptic target in the lateral geniculate nucleus in the thalamus [82]. To recreate this connection, Fligor et al. [83] developed an assembloid model of the visual system where retinal organoids were fused together with cortical and thalamic organoids. They observed robust RGC axonal outgrowth accompanied by an increased RGC survival (up to 150 days) as well as an increase in the retinal organoid size while grown as part of the assembloid compared to control retinal organoids grown on their own [83].

### Direct differentiation via RGC transcription factors

An alternative approach to generate RGCs from hPSCs involves directly programming cells through the overexpression of key transcription factors that drive RGC specification. This strategy introduces intrinsic developmental regulators to accelerate the process, rather than following a stepwise differentiation via extrinsic cues. For example, a single-factor approach using *NGN2* (Neurogenin-2) overexpression, delivered via lentivirus in both hESC and hiPSC, induced rapid neuronal conversion [84]. When combined with a Notch inhibitor DAPT and neurotrophic factors, this yielded RGC-like cells within 10–14 days, with POU4F1 expression observed in 14%−20% of cells by day 7 [84]. Similarly, overexpression of *ATOH7*, *POU4F2*, and *SOX4* induced RGC differentiation, achieving 89.5% POU4F1^+^ cells within 15–20 days when combined with neurotrophic factors such as BDNF and CNTF [85]. Agarwal et al. [86] developed inducible hPSC lines expressing a cocktail of early RGC transcription factors, *NGN2*, *ATOH7*, *ISL1*, and *POU4F2*, collectively termed “*NAIP2*”. Induced expression of these factors, in combination with BMP inhibition, generated RGC-like cells in under one week, with approximately 88–94% of cells identified as POU4F2⁺ RGC neurons [86]. In three independent hPSC lines, this transcription factor combination, delivered with BMP inhibition, consistently produced dense networks of neurite-bearing cells expressing RGC markers [86].

#### Concerns with direct differentiation via RGC transcription factors

Transcription factor mediated programming appears to be the most time-efficient method for generating RGCs from hPSCs. However, it may bypass the early developmental stages of retinal differentiation, including the activation of eye-field and retinal progenitor markers such as *SIX6*, *PAX6*, and *VSX2*. In contrast, conventional differentiation of hPSCs naturally progresses through retinal progenitor and neurogenic stages, enabling the detection of transcriptional differences between disease and control lines [29, 70]. By avoiding these intermediate steps, direct programming may exclude key transcriptional signatures involved in early lineage specification and disease pathogenesis, potentially masking disease phenotypes that only emerge during these transitions.

## Additional enrichment and purification of RGCs

Differentiating hPSCs into RGCs typically generates heterogeneous retinal cell populations. Therefore, additional enrichment steps are often necessary to isolate RGCs for downstream applications. Several purification strategies have been developed, including immuno-based selection methods and physical dissociation approaches. In this section, we review the commonly used techniques: magnetic-activated cell sorting (MACS), fluorescent-activated cell sorting (FACS), immunopanning, and retinal organoid dissociation.

MACS isolates RGCs using magnetic beads conjugated to antibodies targeting surface markers THY1/CD90. Dissociated cells are incubated with antibody-coated magnetic beads, allowing THY1^+^ RGCs bind to the beads. The cell suspension is then passed through a magnetic column, where the labelled RGCs are retained while unlabelled cells flow through. Subsequently, the column is removed from the magnet, and the enriched RGCs are eluted. Studies employing MACS have demonstrated a high level of RGC enrichment, achieving approximately 95% purity in 2D cultures as determined by immunocytochemistry [42], and around 80% from dissociated organoids as assessed by flow cytometry [43, 87]. Even with poor initial differentiation (~ 4% THY1^+^), MACS can enrich RGCs to ~ 77%, based on flow cytometry analysis [45]. This method is time-efficient [88]; however, residual magnetic beads may affect cell function, as observed in other cell types, such as monocytes, where magnetic beads has been shown to impair cell functionality [89].

FACS enables the isolation of RGCs using antibodies against specific surface antigens, most commonly THY1/CD90. In most cases, cells are sorted for sequencing rather than for culture, as FACS imposes significant mechanical and shear stress, adversely affecting post-sort viability [90]. Although FACS can enrich cells to near 100% purity, this comes at the cost of lower yield and reduced survival [90]. FACS has been used to investigate RGC subtypes within THY1^+^ cell populations [3, 47, 91]. However, the process may also induce stress-related and apoptotic gene expression pathways [91], which requires careful interpretation of scRNA-seq data. Additionally, FACS has been applied to enrich cells from dissociated POU4F2-GFP retinal organoids, with the sorted cells subsequently used in transplantation studies in animal models [92].

Immunopanning is another antibody-based technique that captures RGCs on antibody-coated dishes targeting THY1. In this method, petri dishes or flasks are coated with antibodies against RGC markers. Dissociated retinal cells are then added to the coated surfaces, allowing RGCs to bind selectively while non-target cells are washed away. Following incubation, the bound RGCs are gently detached from the surface for collection. A two-step immunopanning protocol has been developed to purify hiPSC-derived RGCs from retinal organoids, achieving over 99.5% purity [93]. This two-step approach first eliminates non-neuronal cells before capturing RGCs, resulting in highly specific isolation and thus higher yield compared to MACS [94]. However, immunopanning is relatively more time-consuming and labour-intensive, as it involves multiple preparation steps, including antibody coating of the dishes, cell incubation, and careful detachment of bound cells [95]. Moreover, non-RGCs such as fibroblast-like cells, also express THY1 [96]. These contaminating cells can bind to the anti-THY1 antibody-coated plates, leading to limited RGC enrichment [41].

Dissociation of retinal organoids is a non-selective method for obtaining RGCs but is commonly used as a preparatory step for subsequent enrichment techniques. Retinal organoids at 4–8 weeks of differentiation are enzymatically dissociated into single-cell suspensions using proteolytic enzymes such as papain [43]. These cells are then plated onto adherent surfaces (coated with laminin) and cultured in media that support RGC survival and maturation – typically supplemented with neurotrophic factors [60, 93]. Although dissociation itself does not selectively isolate RGCs, the combination of RGC-supportive culture conditions and an adherent environment appears to favour their survival and differentiation, particularly when performed at developmental stages where RGCs are predominant within the organoid [43]. This highlights the importance of optimising the timing of dissociation, which may vary depending on the specific organoid protocol. For example, dissociation of retinal organoids at day 30, followed by seven days of adherent culture has been associated with the expression of RGC markers such as POU4F1 and THY1, as detected by immunostaining [97]. Similarly, dissociation at day 40 followed by adherent culture for 80 days has been shown to promote neurite extension and the expression of RGC markers, including POU4F, ISL1, SNCG, and βIII-tubulin [48]. Consistent finding was reported by Rabesandratana et al. [43], who dissociated 30-day-old organoids, replated the cells in 2D and observed that approximately 60% of the dissociated cell population at day 56, following seven days of adherent culture, expressed THY1, which enabled magnetic sorting to enrich RGC populations [43].

Table 2 summarises published protocols (2020–2025) for the differentiation of RGCs from hPSCs. It outlines the culture format, including 2D adherent, 3D organoids and transcription factor-based induction, as well as enrichment or isolation methods, and the percentage of RGC marker expression, as assessed by FACS, immunocytochemistry, or scRNA-seq. The total duration of each protocol is also included.

**Table 2:** Overview of RGC differentiation protocols from hPSCs published in the past five years (2020–2025)

Cell type	Culture format	Isolation/enrichment Method	Percentage of RGCs	Total protocol length	References
hiPSC • keratinocytes or blood cells- derived	2D adherent culture	None	Flow: • 87% POU4F^**+**^ • 93% SNCG^**+**^ • 22.5% RBPMS^**+**^ • 85.5% CD90/THY1^**+**^	36 days	[47]
hiPSC: • CHOPWT8 • CHOPWT9 • CHOPWT10	2D adherent culture	MACS using Thy1.2 microbeads	Flow (before MACS): • 58% THY1^+^ • 84% POU4F2^+^ • 12% RBPMS^+^ Immunocytochemistry: • 95% POU4F1^+^ (with MACS)	35 days	[42]
hiPSC	2D adherent culture	None	Varies between hiPSC-derived RGCs from different patients Flow: • 49.5–87% POU4F^+^ • 6–93% SNCG^+^ • 2.3–46% RBPMS^+^ • 42–91%CD90/THY1^+^	36 days	[60]
hiPSC • SIX6 risk allele • Healthy control	2D adherent culture of neural rosettes to RGCs	None	scRNA seq: • 11.6% mature RGC (58% on retinal lineage; 20% of those in mature RGC stage)	39 days	[29]
hiPSC • *MT-ND4* mutation • Healthy control	3D embryoid bodies and neurospheres + 2D adherent culture of plated neurospheres	None	Not stated	27 days for embryoid bodies, neurosphere generation + 18 days for neurosphere plating to neurite outgrowth	[98]
hiPSC • healthy control; fibroblast- and Müller glia-derived	3D retinal organoids + 2D adherent culture of dissociated, enriched cells	• Dissociation of retinal organoids • MACS using human THY1 antibody	Flow: • 60% THY1⁺ cells before sorting (dissociated culture) • 78% THY1⁺ with MACS enrichment	56 days for retinal organoid generation + 7 days for RGC adherent culture	[43]
hESC: • H1	3D retinal organoids + 2D adherent culture of dissociated cells	Dissociation of retinal organoids	Immunocytochemistry: • 50% ATOH7^+^ • 30% SNCG^+^ • 30% ISL1^+^ • 50% HuC/D^+^	28 days for retinal organoid generation + 10 days for RGC adherent culture	[24]
hiPSC • TLHD2 • 201B7	3D retinal organoids + 2D adherent culture of dissociated, enriched cells	• Dissociation of retinal organoids • Two-step immunopanning	Not stated	55–65 days for retinal organoid generation + 3–5 days for RGC adherent culture	[93]
hiPSCs • corneal keratocytes-derived	3D retinal organoids + 2D adherent culture of dissociated cells	Dissociation of retinal organoids	Not stated	30 days for retinal organoid generation	[97]
hPSC: • *OPTN*(E50K) mutation	3D retinal organoids + 2D adherent culture of dissociated, enriched cells	• Dissociation of retinal organoids • MACS using Thy1.2	Immunocytochemistry: • 20–35% POU4F^+^ • 15–30% ISL1^+^	45 days for retinal organoid generation + up to 28 days for RGC adherent culture	[99]
hiPSC: • fibroblast-derived	3D retinal organoids + 2D adherent culture of retinal organoids	• None	scRNA-seq: 17% RGCs (based on the expression of POU4F2, ISL1, RBPMS, SNCG, GAP43, NEFL/M, ELAVL4, EOMES, and DCX)	35 days for retinal organoid generation + 14 days for retinal organoid adherent culture	[70]
hiPSC • MT-ND4 mutation • Healthy control	3D retinal organoids + 2D adherent culture of retinal organoids	• None	Not stated	35 days for retinal organoid generation + 17 days for retinal organoid adherent culture	[100]
hiPSC: • IMR90.4 • GM23720 hESC • WA09	2D adherent culture Genome-integrated transcription factors: *NGN2*,* ATOH7*,* ISL1*,* POU4F2*	• Clonal selection with zeocin	Immunocytochemistry: 88–94% POU4F2^+^ (tdTomato^+^)	6–7 days	[86]
hiPSC: • 297-1 hESC: • POU4F2-driven mCherry H7-A81	2D adherent culture Lentiviral delivery of transcription factors: *NGN2*	• Selection with puromycin	Immunocytochemistry: 14–20% POU4F1^+^	7 days	[84]
hiPSC: • NTUH-iPSC-02-02 • NTUH-iPSC-01–05	2D adherent culture Lentiviral delivery of transcription factors: *ATOH7*,* POU4F2*,* and SOX4*	• Selection with puromycin + zeocin	Immunocytochemistry: • 89.5% POU4F1^+^ • 94.1% ISL1^+^ 85.3% POU4F1^+^/ISL1^+^	15–20 days	[85]

## Cryopreservation and biobanking of RGCs

Cryopreservation of hPSC-derived RGCs represents a critical step towards scalable production and long-term storage for both regenerative medicine and drug discovery. Early work by Sucher et al. (1991) showed that postnatal rat RGCs could be frozen using DMSO-based protocols without losing viability or function, while maintaining normal ionic conductance [101]. This study demonstrated that neurons such as RGCs can survive freezing and thawing, although it assessed only short-term survival over 48 h and did not examine long-term health or synaptic function, which are important for translational applications. Building on this foundation, recent studies explored cryopreservation of hPSC-derived RGCs. Freezing isolated RGCs from dissociated retinal organoids led to major cell loss, with viability dropping to about 51% in unsorted cells and 25% in purified THY1^+^ cells [43]. In contrast, cryopreserving RGCs within retinal organoids [43] or as progenitor populations [61] produced higher viable yields.

Hence, these findings suggest that cryopreservation success depends on both the developmental stage and format of cells, with storing RGCs within organoids or as expandable retinal progenitor cells currently offering the most reliable method.

## Functional assays for hPSC-derived RGCs

Electrophysiological recording remains the gold standard for assessing neuronal functionality, as neurons are intrinsically electrically excitable and communicate via electrical signals [102]. Patch-clamp and multi-electrode recordings provide direct, high-resolution measurements. hPSC-derived RGCs exhibit voltage-gated sodium and potassium currents and fire action potentials, suggesting they display fundamental properties of neurons [42, 43, 99, 103]. These assays can even detect synaptic events: hPSC-RGCs respond to glutamate with inward currents, indicating the presence of functional synaptic receptors [43]. However, this technique is low-throughput and technically demanding, requiring specialised equipment and expertise.

Calcium imaging provides a non-invasive, optical method for monitoring the activity of many neurons simultaneously [104]. Intracellular calcium levels correlate with neuronal firing and can be visualised in real time using calcium-sensitive dyes [105]. For example, hESC-derived RGCs display calcium responses to GABA (γ-aminobutyric acid, an inhibitory neurotransmitter), reflecting immature RGC physiology [106]. hiPSC-derived RGCs generated via overexpression of *ATOH7*, *POU4F2*, and *SOX4*, demonstrated both stimulus-induced calcium influx and spontaneous calcium transients, confirming the presence of functional voltage-gated calcium channels [85]. However, compared to electrophysiological recordings, calcium imaging is an indirect and relatively slow measure of electrical activity, as calcium transients lag behind rapid voltage changes [102, 107]. Furthermore, calcium signals generally report only suprathreshold activity and do not capture subthreshold potentials [105]. Hence, this technique is best suited for assessing relative activity patterns rather than providing precise electrophysiological measurements.

RGCs extend long axons and rely on efficient movement of organelles (especially mitochondria) to meet energy demands. Axonal transport is vital for RGC health and has been described in hPSC-derived RGCs, with comparable rate to that observed in mature neurons [45]. This functional assessment is technically demanding and requires axon clarity, which dense or tangled neurite networks can complicate tracking [108, 109].

Functional maturity of RGCs can also be assessed by examining their ability to form synaptic connections, as it is a hallmark of mature RGCs [110]. In 2D cultures of RGCs derived from dissociated retinal organoids, these cells have been shown to develop synaptic contacts, evidenced by a significant increase in synaptic markers along their neurites, and higher expression of synaptic proteins over time, including Synapsin-1, vGlut2, Neurexin-1, and SNAP25 [111]. Retinal organoids cultured to ~ 200 days can develop inner retinal circuits, such as ribbon synapses that connect photoreceptors in the outer nuclear layer with bipolar and horizontal cells in the inner nuclear layer [51, 80]. However, there are some limitations when assessing synaptic connectivity. Firstly, immunostaining and protein detection of synaptic markers are indirect methods and cannot conclusively demonstrate functional synaptic activity. Secondly, although late-stage organoids develop mature ribbon synapses by approximately day 200, the RGC population has typically declined by this stage, comprising only about 0.36% of total organoid cells within 168–266 days in one study [112], which limits the stability and duration of functional inner retinal circuits. Nevertheless, several studies have shown that human retinal organoids can exhibit light-evoked responses, consistent with the presence of functional circuitry between photoreceptors, bipolar cells, and RGCs. In retinal organoids aged 150–180 days, light-driven spiking activity has been detected using multielectrode array (MEA) recordings [113], including evidence of both ON and OFF RGC responses [114]. Complementary approaches, such as calcium imaging combined with MEA recordings, have further demonstrated that these light responses are glutamate-dependent and involve RGCs within the organoid structure [112].

## Translational implications of hPSC-derived RGCs

**Fig. 3:**
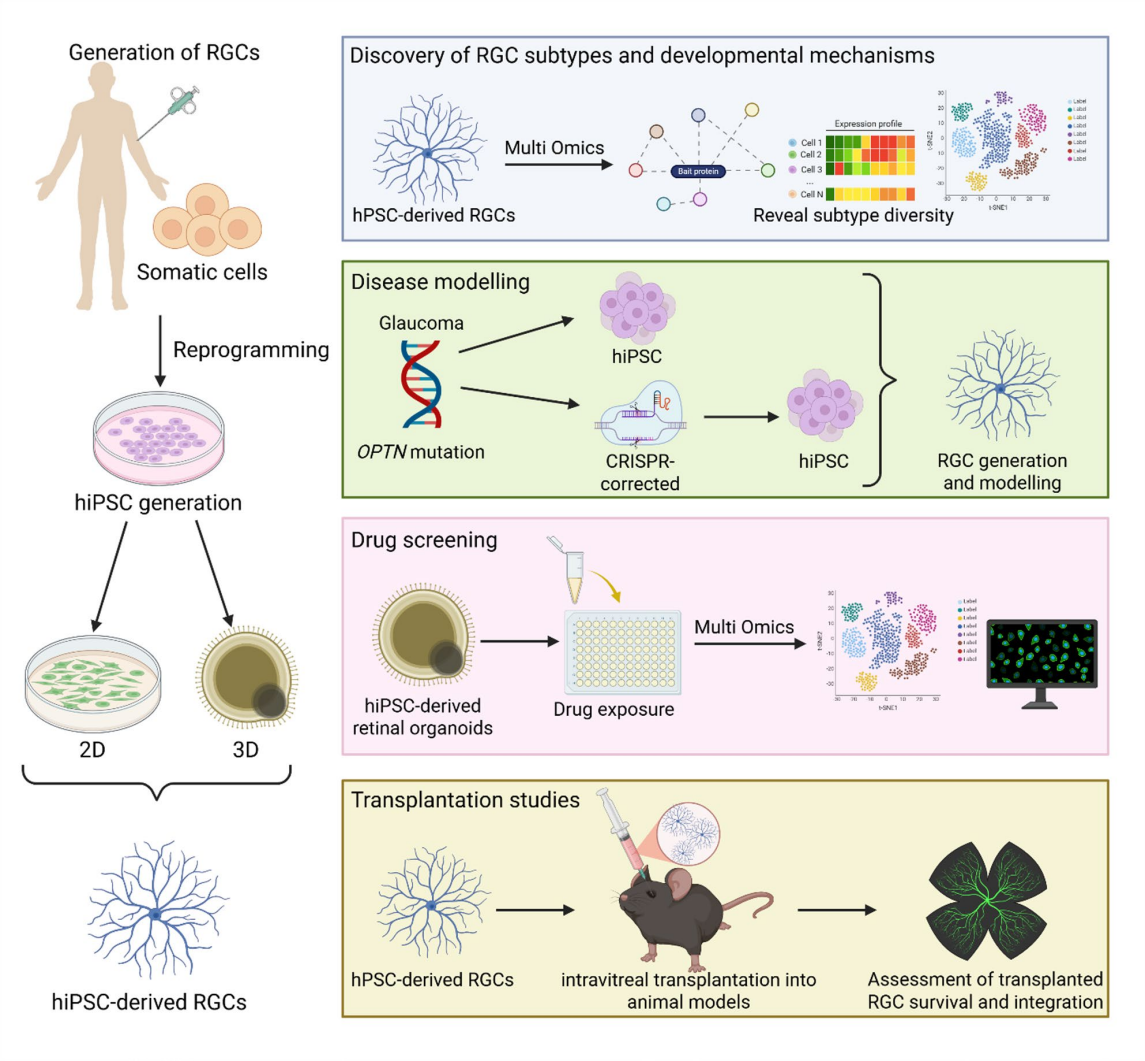
Translational applications of hiPSC-derived RGCs. Schematic overview of the generation of RGCs from somatic cells via reprogramming into hiPSCs and subsequent differentiation using 2D or 3D methods. hiPSC-derived RGCs can be applied to (i) discovery of RGC subtypes and developmental mechanisms through multi-omics profiling, (ii) disease modelling using patient-specific or CRISPR-corrected lines, (iii) drug screening for retinal toxicity and neuroprotection, and (iv) transplantation studies assessing donor RGC survival, integration, and function in animal models. Figure created with BioRender.com

Beyond their molecular and functional validation, hPSC-derived RGCs serve as a versatile platform for translational research. Their applications span from uncovering RGC subtype diversity to modelling optic neuropathies, screening therapeutic compounds, and exploring transplantation strategies for vision restoration (Fig. 3).

### Comparison of gene expression profiles in 2D vs. 3D-derived rgcs: implications for subtype discovery

Single-cell atlases have greatly advanced our knowledge of human RGCs but remain incomplete in resolving subtype diversity. The Human Retina Cell Atlas (HRCA; over 2 million cells/nuclei from 52 donors) provides a multimodal reference, yet its overview emphasises major retinal classes rather than a fully resolving RGC diversity as RGC nuclei represent only ~ 5% of the total detected [115]. Yan et al. [116] profiled ~ 85,000 cells and, using THY1 enrichment, increased the < 2% of RGC fraction to identify 12 RGC types, including midget, parasol, and rare ipRGCs. In contrast, earlier unbiased atlas by Lukowski et al. [117] and Wang et al. [118] confirmed RGC identity but collapsed them into a single cluster due to low yields and insufficient power to distinguish subtype-specific transcriptomic differences. These limitations reflect the rarity and fragility of RGCs, leaving ultra-rare types under-sampled.

hPSC-derived RGC models help address this challenge by providing scalable populations for scRNA-seq, enabling enrichment and targeted discovery of rare or transient subtypes. To enable robust cross-platform comparisons between tissue-derived and hPSC-derived RGCs, it will be essential to establish a consistent set of molecular markers for defining RGC subtypes. The types of RGCs reported from HRCA and hPSC-derived RGC models are summarised in Table 3. The human atlas primarily focuses on common RGC types, including midget and parasol, whereas hPSC-derived RGC models often do not specify or quantify these abundant populations. Instead, they highlight several additional subtypes of RGCs that are molecularly categorised but remain functionally uncharacterised in the human retina.

**Table 3:** Summary of RGC subtypes identified in human retinal atlases and hPSC-derived RGC models

Source	RGC Subtypes Identified
Adult human retina [115, 116]	1. Off midget RGC (*TBR1*) 2. ON midget RGC (*TPBG*) 3. OFF parasol RGC (*FABP4*) 4. ON parasol RGC (*CHRNA2*) 5. ipRGC (*OPN4*) 87.9% midget RGCs, 1.8% parasol RGCs [115] 86% midget RGCs, 10% parasol RGCs [116]
2D hiPSC differentiation [47, 91]	1. ON-OFF direction-selective RGCs (*DCX*) 2. J-RGCs (*JAM2*) 3. some ON-sustained/“alpha” traits (*SPP1*, *FSTL4*) 4. ipRGCs (*OPN4*)
hPSC-derived retinal organoids, dissociated on day 40, and cells were replated in 2D format for 80 days [48]	1. ON-OFF direction-selective-RGCs (*CDH6*) 2. ON- direction-selective RGCs (*FSTL4*) 3. ipRGCs (*OPN4*) 4. alpha RGCs (*SPP1*, *KCNG4*) 5. PV-RGCs (*PVALB*) 6. W3B-RGCs (*SDK2*) 7. J-RGCs (*JAM2*)
Transcription factor-based programming [86]	1. alpha RGCs (*SPP1*,* PIGO*) 2. direction-selective-RGC markers (*DCX*,* JAM2*,* GSN*) 3. *OPN4* not detected, ipRGCs identified via label transfer approach 40% midget RGCs, 13% parasol RGCs, 7% ipRGCs, 40% other poorly resolved RGC subtypes

2D differentiation produced hiPSC-derived RGCs that expressed canonical RGC markers (*BRN3A/POU4F1*,* BRN3B/POU4F2*,* ISL1*,* RBPMS*,* TUBB3*,* MAP2*, and *SNCG*) by day 40 using a rapid dual-SMAD/Wnt inhibition protocol [47]. scRNA-seq revealed multiple distinct clusters, ranging from cells expressing retinal progenitor markers such as *SFRP2* to those enriched for neuronal differentiation and axon guidance genes such as *GAP43* and *DCC*, indicating heterogeneity in maturation and a progressive shift towards mature RGC phenotypes [47]. Our scRNA-seq of THY1^+^ RGCs FACS-sorted from an hESC POU4F2-mCherry reporter line (A81-H7) identified three RGC clusters with varying maturity. The most mature cluster upregulated axon guidance and synaptic genes such as *MAPK10* and downregulation of cell cycle genes such as *MYC*, indicating postmitotic maturation [91]. Even in 2D culture, hPSC-derived RGCs displayed molecular signatures of diverse subtypes, including ON–OFF direction-selective RGCs (*DCX*), J-RGCs (*JAM2*), and ON-RGCs marked by *FSTL4* and *SPP1* [47]. Intrinsically photosensitive RGCs (ipRGCs), marked by O*PN4*, were also detected [91]. Overall, 2D differentiation yields multiple subtype-like identities, but specialised subtypes may require additional cues to develop or may only appear at very low frequency.

In retinal organoids, gene expression analyses are more complex as they contain all major retinal cell types. Nonetheless, organoids provide a closer approximation to the native developmental context, as RGCs form alongside other retinal neurons, allowing nascent inner retinal layers and more physiologically relevant cell-cell interactions. Early-stage organoid-derived RGCs (~ day 40) expressed canonical RGC markers as seen in 2D cultures (*POU4F*, *ISL1*, *RBPMS*, and *SNCG*), confirming that RGC identity is achieved in both systems [26, 48]. scRNA-seq at 12 weeks (84 days) of development identified two major RGC populations distinguished by *POU4F1* expression levels, and further sub-clustering revealed 11 transcriptionally distinct RGC clusters including one with an apoptotic signature, suggesting that some subtypes are especially vulnerable during maturation [119]. It suggests that certain RGC subtypes may be more vulnerable over time, an important consideration for modelling neurodegenerative diseases like glaucoma in organoids. Organoids support broader subtype diversity than 2D cultures, including ON-OFF direction-selective RGCs (e.g. *CDH6*), ON direction-selective RGCs (e.g. *FSTL4*), ipRGCs (e.g. *OPN4*), alpha RGCs (high expression of *SPP1*, *CB2*, and *KCNG4*) and several rare RGC subtypes including PV-, W3B-, and J-RGCs, identified by *PVALB*, *SDK2*, and *JAM2*, respectively [48]. Incorporating developmental timing enables tracking of sequential subtype emergence, which is valuable for modelling differential susceptibility of RGCs to injury and disease [120].

Transcription factor-based programming generates induced RGCs that express canonical RGC markers (e.g. *POU4F1/2*,* ISL1*,* RBPMS* and *SNCG*) and subtype specific markers, including for alpha RGCs (*SPP1*, *PIGO*, *RPP13*) and direction-sensitive RGCs (*DCX*, *CDH6*, *HAM2*, *GSN*) [86]. Some subtype markers (such as *JAM2* and *GSN*) showed increased expression over time, which could indicate a growing proportion of direction-sensitive RGCs; and some subtype specific markers can be co-expressed, reflecting the immature or transitional state of some induced RGCs. It is noteworthy that melanopsin-expressing ipRGCs (OPN4⁺) were essentially absent in the induced RGC populations [86].

#### Limitations of RGC subtype classification with transcriptomic analysis

Primate retinae, including humans, have long been classified by anatomical and physiological features, with approximately 18 RGC types defined based on distinct structural and functional characteristics [1, 121]. Anatomical classification typically relies on soma size, dendritic field diameter and shape, and the laminar stratification of dendrites within the inner plexiform layer, which together reflect each cell’s visual processing role [1]. However, dendritic and somatic architecture can vary significantly with retinal eccentricity, i.e., distance from the fovea toward the retinal periphery [122], developmental stage [123], and species [121]. This structural variation makes it difficult to apply consistent anatomical definitions of RGC subtypes across different species, ages, or retinal regions. In primate retinae, the most predominant classes are the ON and OFF midget RGCs, which together account for ~ 80% of all human RGCs by morphological criteria, followed by the ON and OFF parasol RGCs, comprising ~ 10% [116]. Midget RGCs have small dendritic fields and sustained, colour-opponent (red-green) responses and low contrast gain [1, 124], whereas parasol RGCs possess large dendritic fields and transient, achromatic responses optimised for high temporal contrast and high contrast gain [125, 126]. Other classical types include small bistratified (blue-yellow colour-opponent) RGCs, wide-field and orientation-selective RGCs, and intrinsically photosensitive (ipRGC) [4]. However, these anatomical and functional categories were defined long before modern single-cell transcriptomic profiling, and do not always map one-to-one onto molecularly defined classes. Recent single-cell RNA atlases in primates have identified up to 18 transcriptionally distinct RGC clusters in primates [4], but their correspondence to canonical morphological types such as midget and parasol cells remains unresolved, and inferred indirectly through abundance and comparative analyses with the macaque data [116]. Moreover, overlapping gene-expression profiles can obscure distinctions. For instance, all ipRGCs express OPN4, yet this molecular signature encompasses multiple functional subclasses in mice [127]. A unified understanding of human RGC diversity therefore requires multimodal integration, linking morphology, physiology, and gene expression. A modified Patch-seq approach demonstrated that it is possible to collect molecular, electrophysiological, and morphological data from individual mouse RGCs, and align each cell’s transcriptome with known gene-expression clusters [128]. Although this approach cannot be applied to live human retina because of limited tissue accessibility, it offers a practical framework for new cell-type discovery using hPSC-derived RGC systems.

### hPSC-derived RGCs for disease modelling

RGCs are affected in several optic neuropathies. Some recent examples are given below to illustrate the use of hPSC-derived RGCs in modelling of these conditions.

#### Glaucoma

Glaucoma, a leading cause of irreversible blindness, is a third most heritable human disease [8, 129]. A recent genome-wide association study (GWAS) identified over 100 additional risk loci for glaucoma, increasing the number of independent risk loci to 312 [130]. One of the variants identified by the GWAS lies near Optineurin (*OPTN*) gene which has previously been associated with glaucoma [131]. The most common *OPTN* mutation is the Glu50Lys (E50K). The mutation causes fragmentation of the Golgi apparatus and impair vesicular transport [132]. It also disrupts *OPTN* translocation from the Golgi apparatus to the nucleus, thereby preventing cytochrome c release from mitochondria, which normally protects cells from H_2_O_2_-induced oxidative stress [133].

hiPSC-derived RGCs have proven valuable for modelling glaucoma-related pathogenesis. Studies using hiPSC-derived RGCs carrying the OPTN (E50K) mutation have revealed disease-relevant phenotypes in both 3D retinal organoids and 2D cultures [99, 134, 135]. Notably, VanderWall et al. [99] generated patient-derived and CRISPR-corrected isogenic hiPSC lines, showing that while both formed retinal organoids efficiently, the mutant lines displayed impaired autophagy and elevated apoptosis. In 2D, mutant RGCs exhibited neurodegenerative features, including neurite shortening and transcriptomic signatures indicating disrupted protein clearance and axonal maintenance. Building on their initial work, the same group used 2D cultures of OPTN (E50K) hiPSC-derived RGCs and isogenic controls to investigate neuron-glia interactions [134] and autophagy dysfunction [135]. In co-culture studies, RGCs exhibited hyperexcitability when grown with OPTN-mutant astrocytes, a phenotype alleviated by healthy astrocyte co-culture [134]. Mutant astrocytes secreted lower levels of IL-6 and IL-8, and exogenous IL-6 supplementation improved RGC morphology, underscoring the importance of astrocyte-derived cues. In parallel, Huang et al. [135] demonstrated that OPTN (E50K) RGCs showed autophagy deficits linked to AMPK activation and mTORC1 inhibition, which were worsened under stress conditions such as insulin deprivation. Treatment with the autophagy enhancer trehalose rescued neurite outgrowth, highlighting autophagy modulation as a potential therapeutic strategy [135].

Given the polygenic nature of glaucoma, large-scale in vitro models are necessary to uncover low-penetrance risk variants. In our recent study, we generated retinal organoids from 110 hiPSC lines derived from both healthy individuals and those with primary open-angle glaucoma, and performed scRNA-seq on over 247,000 cells [70]. This analysis identified 4,443 eQTLs, including 312 specific to RGCs, with 97 associated with glaucoma. Notably, *KANSL1-AS1* emerged as a novel glaucoma risk locus [70], highlighting the utility of scalable hPSC-derived RGC models for mapping gene regulation and discovering disease modifiers relevant to precision medicine.

#### Leber’s hereditary optic neuropathy (LHON)

LHON is caused by mitochondrial DNA mutations, most commonly m.11778G > A (*MT-ND4*), m.3460G > A (*MT-ND1*), and m.14484T > C (*MT-ND6*), which impair oxidative phosphorylation and compromise energy production in RGCs. Patient-specific hiPSC-derived RGCs carrying *MT-ND1* and *MT-ND6* mutations demonstrated increased apoptosis [136]. Similar approaches using *MT-ND4*-mutant lines revealed elevated reactive oxygen species, apoptosis, and upregulation of the pro-apoptotic circular RNA *hsa_circ_0087207* [98]. RGCs derived from *MT-ND4* mutant hiPSCs also showed reduced expression of key RGC markers (*POU4F1*, *SNCG*, *ATOH7*), impaired differentiation, and transcriptomic signatures indicating dysregulation of N6-methyladenosine (m6A) RNA methylation, including reduced levels of METTL3 and YTHDF1 [100]. Together, these findings reinforce the utility of hPSC-derived RGCs in modelling LHON-associated pathologies, such as energy failure, oxidative stress, and apoptosis, while also revealing novel regulatory mechanisms, including circRNA activity [98] and epitranscriptomic imbalance [100], that may offer therapeutic potential.

#### Autosomal dominant optic atrophy (ADOA)

ADOA, marked by early-onset visual acuity loss, is primarily driven by mutations in *OPA1* that impair mitochondrial dynamics [137, 138]. RGCs differentiated from *OPA1*-mutant displayed fragmented mitochondrial networks and impaired mtDNA maintenance, indicating underlying metabolic vulnerability [139]. In contrast, RGCs harbouring CRISPR-engineered *OPA1* mutations exhibited intrinsic differentiation impairments, including smaller soma size, reduced neurite length, downregulation of *POU4F2* and *NEFL*, altered electrophysiology, and decreased progenitor proliferation [140]. Mitochondrial dysfunction was a consistent feature across both models [139, 140].

#### Limitation in disease modelling with hiPSC-derived RGCs

While hiPSC-derived RGCs and retinal organoids provide powerful platforms for dissecting the genetic architecture of optic neuropathies, these models cannot fully recapitulate the complex gene-environment interactions that contribute to disease onset and progression in vivo. In glaucoma, for example, elevated IOP, oxidative stress, and aging interact to induce mechanical and metabolic strain on RGCs. These factors trigger neurodegenerative cascades involving mitochondrial dysfunction, inflammation, and cellular senescence [141, 142], which are challenging to model in standard cultures. Similarly, in LHON, risk factors such as vitamin B_12_ deficiency [143] and chronic alcohol consumption [144] can exacerbate mitochondrial stress and RGC vulnerability. These conditions are not readily captured in current in vitro systems. In ADOA, age-related declines in OPA1 level reduce mitochondrial reserve in older individuals, making them more susceptible to neurodegeneration [145]. These examples highlight a key limitation of current 2D and organoid models. Although genetically tractable, they often lack the ability to reflect environmental influences on disease. Incorporating biomechanical or microfluidic systems that mimic ocular pressure, oxidative load, or nutrient deprivation, along with extended culture to model ageing, may help address this gap and improve the translational relevance of hiPSC-derived models.

### hPSC-derived RGCs for drug screening

hiPSC-derived RGCs represent a promising platform for assessing drug efficacy and toxicity in a patient-specific context, enabling personalised therapeutic strategies. Although the application of these models for drug screening is still in its early stages, several studies have demonstrated their potential in evaluating retinal toxicity and identifying neuroprotective compounds.

Long-term differentiated retinal organoids (143–199 days) were exposed to five compounds with known retinal toxicity (digoxin, thioridazine, sildenafil, ethanol, methanol) as well as the anti-inflammatory agent ketorolac as a control [146]. After dose titration and cytotoxicity screening using an LDH assay, organoids underwent confocal and electron microscopy, scRNA-seq, and electrophysiological profiling via multielectrode arrays. Exposure to digoxin and thioridazine resulted in significant loss of multiple retinal cell types, including RGCs, and induced astrocyte activation and caspase-3 apoptosis, a phenotype also observed with ethanol. Notably, ketorolac, despite lacking structural toxicity, modulated expression of genes related to the unfolded protein response, revealing subtle transcriptomic effects in line with its known ocular side effects [146].

In a model of ethambutol-induced optic neuropathy, induced RGC-like cells generated through transcription factor programming were exposed to the anti-tuberculosis drug [85]. Treatment triggered dose- and time-dependent increases in apoptosis and neurite degeneration. Mechanistically, ethambutol impaired autophagic flux, as indicated by p62 and LC3-II accumulation, and elevated intracellular ROS levels. Co-treatment with N-acetylcysteine reduced oxidative stress and partially rescued cell survival and neurite integrity, implicating oxidative damage as a key mediator of RGC toxicity [85].

To simulate axonal stress, transcription factor-induced RGCs were treated with colchicine (a microtubule destabiliser) and paclitaxel (a stabiliser), mimicking injury conditions [86]. Kinase inhibitors GNE-3511 and PF-06260933 were then evaluated for neuroprotection. GNE-3511 inhibited DLK/LZK signalling and preserved both cell survival and neurite structure under colchicine-induced stress. PF-06260933, targeting the GCK-IV pathway, conferred protection against paclitaxel toxicity and significantly improved RGC survival across multiple hiPSC lines. Notably, GCK-IV inhibition also promoted robust neurite outgrowth in the absence of injury, suggesting potential for axonal regeneration strategies [86].

Finally, in a glaucoma-relevant model, RGCs derived from retinal organoids carrying the *OPTN* E50K mutation exhibited progressive neurite retraction, protein aggregate accumulation, impaired autophagic flux, and reduced mTORC1 activity. Treatment with trehalose, an mTOR-independent autophagy enhancer, restored autophagy, reduced aggregate load, and rescued neurite degeneration, offering a potential therapeutic avenue for glaucoma-linked RGC degeneration [135].

Together, these studies demonstrate the utility of hPSC-derived RGCs for modelling drug responses and mechanistic toxicity, supporting their future application in neuroprotective screening pipelines and personalised medicine.

### hPSC-derived RGCs in transplantation studies

hPSC-derived RGCs have been explored for cell replacement therapies in optic neuropathies, using both 2D and 3D differentiation protocols. While early studies demonstrated donor cell survival following intravitreal injection, they also highlighted critical limitations, including low integration efficiency, incomplete maturation, and lack of neurite outgrowth. For instance, hESC-derived RGC-like cells transplanted into healthy rat eyes survived and expressed RGC markers but remained immature and failed to extend processes or integrate into host circuitry [106]. Advancements in 2D protocols have since improved outcomes. hiPSC-derived RGCs expressing SNCG-eGFP were transplanted intravitreally into immunocompetent mice and survived for at least five months in a subset of animals [60]. Some donor cells integrated into the ganglion cell layer and exhibited neuronal morphology and electrophysiological responsiveness, though overall survival remained low, and the absence of injury may have limited engraftment [60]. Organoid-based protocols have also been employed, often incorporating purification steps such as FACS or MACS to enrich for RGC populations prior to transplantation [43, 92]. In optic nerve injury models, purified hiPSC-RGCs survived for several weeks, expressed canonical RGC markers, and in some cases extended axons aligned with host nerve fibre bundles. Integration and survival were enhanced in injured retinas, suggesting that retinal damage creates a more permissive environment for donor incorporation [43, 92]. Transplantation of less differentiated retinal progenitors has also shown promise. hiPSC-derived progenitor cells injected into NMDA-injured mouse eyes migrated to the ganglion cell layer, expressed early neuronal markers, and extended axon-like processes. However, functional restoration was not achieved, and long-term integration remains unproven [147]. Collectively, these studies demonstrate that hPSC-derived RGCs and progenitors can survive, express relevant markers, and exhibit limited structural and functional integration after transplantation. Critically, outcomes are improved in injury models, underscoring the importance of the host microenvironment. While promising, further optimisation is required to enhance donor cell maturation, purity, axon guidance, synaptic connectivity, and immune compatibility to support clinically effective vision restoration.

### Challenges in applying hPSC-derived RGCs for translational purposes

Despite significant advances, several major obstacles hinder the routine use of hPSC-derived RGCs in disease modelling, drug screening, and cell replacement therapies. A key limitation is the lack of standardised differentiation protocols. Diverse methods yield RGCs with varying efficiencies, maturational states, and subtype compositions. Given the functional diversity of RGC subtypes, it remains unclear which subtypes are most disease-relevant, complicating cross-study reproducibility. Moreover, hPSC-derived RGCs often exhibit immature phenotypes lacking adult features, potentially limiting their capacity to model late-onset disease mechanisms [148]. Scalability is another barrier, particularly for drug screening and population-scale disease modelling. Generating large numbers of high-quality RGCs from multiple patient lines is time-intensive and costly. Variability in culture conditions and genetic background introduces noise that can obscure disease-specific signatures, and most assays rely on molecular markers or survival endpoints rather than functional metrics such as axonal transport or synaptic activity. The absence of standardised functional readouts further reduces inter-laboratory comparability.

In transplantation, the challenges are even greater. Donor RGCs show poor survival and limited integration, with most failing to form connections with host neurons or extend axons through the optic nerve, an essential requirement for visual restoration [43, 60, 92, 106, 147, 149]. The degenerating host retina presents a hostile environment marked by glial scarring, inflammation, and loss of trophic support, all of which limit graft viability [5, 150]. Immune rejection also remains a concern. While the eye is partially immune privileged, allogeneic grafts may still be targeted, and long-term immunosuppression carries risks [151]. Autologous transplantation could overcome this, but is currently prohibitively expensive and labour-intensive [152].

## Perspectives and conclusions

hPSC technologies have enabled the generation of RGCs through various differentiation platforms, including 2D cultures, retinal organoids, and transcription factor–based programming. Each approach offers unique strengths and limitations, differing in efficiency, maturation, subtype representation, and fidelity to in vivo development. While all platforms produce RGC-like cells expressing core markers, their outputs must be interpreted with caution, as no single method fully recapitulates the complexity of native RGC development. Multi-marker validation and context-aware model selection remain essential. Despite these limitations, hPSC-derived RGCs have proven to be valuable tools for disease modelling and drug discovery. Studies across glaucoma, LHON, and ADOA have successfully captured genetic and metabolic defects, identified pathogenic pathways, and revealed candidate therapeutic compounds. Moreover, transplantation studies have demonstrated that donor RGCs can survive and integrate into injured retinas, albeit with limited functional connectivity and without long-range axonal regeneration. Looking ahead, the integration of 2D and 3D culture systems with direct programming, single-cell transcriptomics, and functional assays is steadily advancing the field toward a comprehensive molecular atlas of human RGC subtypes. These developments are accelerating both mechanistic insight and therapeutic innovation, while laying the foundation for future regenerative strategies aimed at restoring vision in optic neuropathies.

## Data Availability

No datasets were generated or analysed during the current study.

## Abbreviations

RGC: Retinal ganglion cell
hPSC: Human pluripotent stem cells
IOP: Intraocular pressure
hESCs: Human embryonic stem cells
hiPSCs: Human induced pluripotent stem cells
3D: Three-dimensional
2D: Two-dimensional
ATOH7: Atonal BHLH Transcription Factor 7
POU4F: POU Class 4 Homeobox
ISL1: ISL LIM homeobox 1
SNCG: γ-Synuclein
RBPMS: RNA-binding protein with multiple splicing
NEFM: Neurofilament medium chain
NEFH: Neurofilament heavy chain
BDNF: Brain-derived neurotrophic factor
CNTF: Ciliary neurotrophic factor
scRNA-seq: Single-cell RNA sequencing
NGN2: Neurogenin-2
MACS: Magnetic-activated cell sorting
FACS: Fluorescent-activated cell sorting
GABA: γ-aminobutyric acid
MEA: Multielectrode array
HRCA: The Human Retina Cell Atlas
OPTN: Optineurin
GWAS: Genome-wide association study

## References

[1] Kim US, Mahroo OA, Mollon JD, Yu-Wai-Man P. Retinal ganglion cells-diversity of cell types and clinical relevance. Front Neurol. 2021;12:661938. 10.3389/fneur.2021.661938. 34093409 10.3389/fneur.2021.661938 PMC8175861

[2] Rheaume BA, Jereen A, Bolisetty M, Sajid MS, Yang Y, Renna K, et al. Single cell transcriptome profiling of retinal ganglion cells identifies cellular subtypes. Nat Commun. 2018;9(1):2759. 10.1038/s41467-018-05134-3. 30018341 10.1038/s41467-018-05134-3 PMC6050223

[3] Tran NM, Shekhar K, Whitney IE, Jacobi A, Benhar I, Hong G, et al. Single-Cell profiles of retinal ganglion cells differing in resilience to injury reveal neuroprotective genes. Neuron. 2019;104(6):1039–55. 10.1016/j.neuron.2019.11.006. e12. 31784286 10.1016/j.neuron.2019.11.006 PMC6923571

[4] Peng YR, Shekhar K, Yan W, Herrmann D, Sappington A, Bryman GS, et al. Molecular classification and comparative taxonomics of foveal and peripheral cells in primate retina. Cell. 2019;176(5):1222–e3722. 10.1016/j.cell.2019.01.004. 30712875 10.1016/j.cell.2019.01.004 PMC6424338

[5] Soucy JR, Aguzzi EA, Cho J, Gilhooley MJ, Keuthan C, Luo Z, et al. Retinal ganglion cell repopulation for vision restoration in optic neuropathy: a roadmap from the RReSTORe Consortium. Mol Neurodegener. 2023;18(1):64. 10.1186/s13024-023-00655-y. 37735444 10.1186/s13024-023-00655-y PMC10514988

[6] Yu M, Lin C, Weinreb RN, Lai G, Chiu V, Leung CK. Risk of visual field progression in glaucoma patients with progressive retinal nerve fiber layer thinning: a 5-year prospective study. Ophthalmology. 2016;123(6):1201–10. 10.1016/j.ophtha.2016.02.017. 27001534 10.1016/j.ophtha.2016.02.017

[7] Vision Loss Expert Group of the Global Burden of, Disease S, Blindness GBD, Vision Impairment C. Global estimates on the number of people blind or visually impaired by glaucoma: A meta-analysis from 2000 to 2020. Eye (Lond). 2024;38(11):2036–46. 10.1038/s41433-024-02995-5. 38565601 10.1038/s41433-024-02995-5 PMC11269708

[8] Tham YC, Li X, Wong TY, Quigley HA, Aung T, Cheng CY. Global prevalence of glaucoma and projections of glaucoma burden through 2040: a systematic review and meta-analysis. Ophthalmology. 2014;121(11):2081–90. 10.1016/j.ophtha.2014.05.013. 24974815 10.1016/j.ophtha.2014.05.013

[9] Luo X, Salgueiro Y, Beckerman SR, Lemmon VP, Tsoulfas P, Park KK. Three-dimensional evaluation of retinal ganglion cell axon regeneration and pathfinding in whole mouse tissue after injury. Exp Neurol. 2013;247:653–62. 10.1016/j.expneurol.2013.03.001. 23510761 10.1016/j.expneurol.2013.03.001 PMC3726550

[10] Agostinone J, Alarcon-Martinez L, Gamlin C, Yu WQ, Wong ROL, Di Polo A. Insulin signalling promotes dendrite and synapse regeneration and restores circuit function after axonal injury. Brain. 2018;141(7):1963–80. 10.1093/brain/awy142. 29931057 10.1093/brain/awy142 PMC6022605

[11] Belforte N, Agostinone J, Alarcon-Martinez L, Villafranca-Baughman D, Dotigny F, Cueva Vargas JL, et al. AMPK hyperactivation promotes dendrite retraction, synaptic loss, and neuronal dysfunction in glaucoma. Mol Neurodegener. 2021;16(1):43. 10.1186/s13024-021-00466-z. 34187514 10.1186/s13024-021-00466-z PMC8243567

[12] Duan X, Qiao M, Bei F, Kim IJ, He Z, Sanes JR. Subtype-specific regeneration of retinal ganglion cells following axotomy: effects of osteopontin and mTOR signaling. Neuron. 2015;85(6):1244–56. 10.1016/j.neuron.2015.02.017. 25754821 10.1016/j.neuron.2015.02.017 PMC4391013

[13] Galatro TF, Holtman IR, Lerario AM, Vainchtein ID, Brouwer N, Sola PR, et al. Transcriptomic analysis of purified human cortical microglia reveals age-associated changes. Nat Neurosci. 2017;20(8):1162–71. 10.1038/nn.4597. 28671693 10.1038/nn.4597

[14] Zhang Y, Sloan SA, Clarke LE, Caneda C, Plaza CA, Blumenthal PD, et al. Purification and characterization of progenitor and mature human astrocytes reveals transcriptional and functional differences with mouse. Neuron. 2016;89(1):37–53. 10.1016/j.neuron.2015.11.013. 26687838 10.1016/j.neuron.2015.11.013 PMC4707064

[15] Bringmann A, Syrbe S, Gorner K, Kacza J, Francke M, Wiedemann P, et al. The primate fovea: structure, function and development. Prog Retin Eye Res. 2018;66:49–84. 10.1016/j.preteyeres.2018.03.006. 29609042 10.1016/j.preteyeres.2018.03.006

[16] Masri RA, Percival KA, Koizumi A, Martin PR, Grunert U. Survey of retinal ganglion cell morphology in marmoset. J Comp Neurol. 2019;527(1):236–58. 10.1002/cne.24157. 27997691 10.1002/cne.24157

[17] Takahashi K, Tanabe K, Ohnuki M, Narita M, Ichisaka T, Tomoda K, et al. Induction of pluripotent stem cells from adult human fibroblasts by defined factors. Cell. 2007;131(5):861–72. 10.1016/j.cell.2007.11.019. 18035408 10.1016/j.cell.2007.11.019

[18] Takahashi K, Yamanaka S. Induction of pluripotent stem cells from mouse embryonic and adult fibroblast cultures by defined factors. Cell. 2006;126(4):663–76. 10.1016/j.cell.2006.07.024. 16904174 10.1016/j.cell.2006.07.024

[19] Huang EJ, Liu W, Fritzsch B, Bianchi LM, Reichardt LF, Xiang M. Brn3a is a transcriptional regulator of soma size, target field innervation and axon pathfinding of inner ear sensory neurons. Development. 2001;128(13):2421–32. 10.1242/dev.128.13.2421. 11493560 10.1242/dev.128.13.2421 PMC2710107

[20] Liang X, Song MR, Xu Z, Lanuza GM, Liu Y, Zhuang T, et al. Isl1 is required for multiple aspects of motor neuron development. Mol Cell Neurosci. 2011;47(3):215–22. 10.1016/j.mcn.2011.04.007. 21569850 10.1016/j.mcn.2011.04.007 PMC3200226

[21] O’Hara-Wright M, Gonzalez-Cordero A. Retinal organoids: a window into human retinal development. Development. 2020;147(24):dev189746. 10.1242/dev.189746. 33361444 10.1242/dev.189746 PMC7774906

[22] Gill KP, Hewitt AW, Davidson KC, Pebay A, Wong RC. Methods of retinal ganglion cell differentiation from pluripotent stem cells. Transl Vis Sci Technol. 2014;3(4):7. 10.1167/tvst.3.3.7. 25774327 10.1167/tvst.3.3.7 PMC4356355

[23] Brzezinski JAt, Prasov L, Glaser T. Math5 defines the ganglion cell competence state in a subpopulation of retinal progenitor cells exiting the cell cycle. Dev Biol. 2012;365(2):395–413. 10.1016/j.ydbio.2012.03.006. 22445509 10.1016/j.ydbio.2012.03.006 PMC3337348

[24] Wagstaff EL, Ten Asbroek A, Ten Brink JB, Jansonius NM, Bergen AAB. An alternative approach to produce versatile retinal organoids with accelerated ganglion cell development. Sci Rep. 2021;11(1):1101. 10.1038/s41598-020-79651-x. 33441707 10.1038/s41598-020-79651-x PMC7806597

[25] Mu X, Klein WH. A gene regulatory hierarchy for retinal ganglion cell specification and differentiation. Semin Cell Dev Biol. 2004;15(1):115–23. 10.1016/j.semcdb.2003.09.009. 15036214 10.1016/j.semcdb.2003.09.009

[26] Sridhar A, Hoshino A, Finkbeiner CR, Chitsazan A, Dai L, Haugan AK, et al. Single-Cell Transcriptomic Comparison of Human Fetal Retina, hPSC-Derived Retinal Organoids, and Long-Term Retinal Cultures. Cell Rep. 2020;30(5):1644–59 e4. 10.1016/j.celrep.2020.01.007. 32023475 10.1016/j.celrep.2020.01.007 PMC7901645

[27] Wang SW, Mu X, Bowers WJ, Kim DS, Plas DJ, Crair MC, et al. Brn3b/Brn3c double knockout mice reveal an unsuspected role for Brn3c in retinal ganglion cell axon outgrowth. Development. 2002;129(2):467–77. 10.1242/dev.129.2.467. 11807038 10.1242/dev.129.2.467

[28] Shi M, Kumar SR, Motajo O, Kretschmer F, Mu X, Badea TC. Genetic interactions between Brn3 transcription factors in retinal ganglion cell type specification. PLoS One. 2013;8(10):e76347. 10.1371/journal.pone.0076347. 24116103 10.1371/journal.pone.0076347 PMC3792956

[29] Teotia P, Niu M, Ahmad I. Mapping developmental trajectories and subtype diversity of normal and glaucomatous human retinal ganglion cells by single-cell transcriptome analysis. Stem Cells. 2020;38(10):1279–91. 10.1002/stem.3238. 32557945 10.1002/stem.3238 PMC7586941

[30] Pan L, Deng M, Xie X, Gan L. ISL1 and BRN3B co-regulate the differentiation of murine retinal ganglion cells. Development. 2008;135(11):1981–90. 10.1242/dev.010751. 18434421 10.1242/dev.010751 PMC2758274

[31] Wu F, Kaczynski TJ, Sethuramanujam S, Li R, Jain V, Slaughter M, et al. Two transcription factors, Pou4f2 and Isl1, are sufficient to specify the retinal ganglion cell fate. Proc Natl Acad Sci U S A. 2015;112(13):E1559-68. 10.1073/pnas.1421535112. 25775587 10.1073/pnas.1421535112 PMC4386335

[32] Subramani M, Van Hook MJ, Ahmad I. Reproducible generation of human retinal ganglion cells from banked retinal progenitor cells: analysis of target recognition and IGF-1-mediated axon regeneration. Front Cell Dev Biol. 2023;11:1214104. 10.3389/fcell.2023.1214104. 37519299 10.3389/fcell.2023.1214104 PMC10373790

[33] Latremoliere A, Cheng L, DeLisle M, Wu C, Chew S, Hutchinson EB, et al. Neuronal-Specific TUBB3 is not required for normal neuronal function but is essential for timely axon regeneration. Cell Rep. 2018;24(7):1865–e799. 10.1016/j.celrep.2018.07.029. 30110642 10.1016/j.celrep.2018.07.029 PMC6155462

[34] Riazifar H, Jia Y, Chen J, Lynch G, Huang T. Chemically induced specification of retinal ganglion cells from human embryonic and induced pluripotent stem cells. Stem Cells Transl Med. 2014;3(4):424–32. 10.5966/sctm.2013-0147. 24493857 10.5966/sctm.2013-0147 PMC3973714

[35] Ferreira A, Caceres A. Expression of the class III beta-tubulin isotype in developing neurons in culture. J Neurosci Res. 1992;32(4):516–29. 10.1002/jnr.490320407. 1527798 10.1002/jnr.490320407

[36] Sharma RK, Netland PA. Early born lineage of retinal neurons express class III beta-tubulin isotype. Brain Res. 2007;1176:11–7. 10.1016/j.brainres.2007.07.090. 17900541 10.1016/j.brainres.2007.07.090

[37] Ekstrom P, Johansson K. Differentiation of ganglion cells and amacrine cells in the rat retina: correlation with expression of HuC/D and GAP-43 proteins. Brain Res Dev Brain Res. 2003;145(1):1–8. 10.1016/s0165-3806(03)00170-6. 14519488 10.1016/s0165-3806(03)00170-6

[38] Surgucheva I, Weisman AD, Goldberg JL, Shnyra A, Surguchov A. Gamma-synuclein as a marker of retinal ganglion cells. Mol Vis. 2008;14:1540–8. 18728752 PMC2518532

[39] Yang Q, Liu L, He F, Zhao W, Chen Z, Wu X, et al. Retinal ganglion cell type-specific expression of synuclein family members revealed by scrna-sequencing. Int J Med Sci. 2024;21(8):1472–90. 10.7150/ijms.95598. 38903914 10.7150/ijms.95598 PMC11186421

[40] Martersteck EM, Hirokawa KE, Evarts M, Bernard A, Duan X, Li Y, et al. Diverse central projection patterns of retinal ganglion cells. Cell Rep. 2017;18(8):2058–72. 10.1016/j.celrep.2017.01.075. 28228269 10.1016/j.celrep.2017.01.075 PMC5357325

[41] Sluch VM, Chamling X, Liu MM, Berlinicke CA, Cheng J, Mitchell KL, et al. Enhanced stem cell differentiation and immunopurification of genome engineered human retinal ganglion cells. Stem Cells Transl Med. 2017;6(11):1972–86. 10.1002/sctm.17-0059. 29024560 10.1002/sctm.17-0059 PMC6430043

[42] Chavali VRM, Haider N, Rathi S, Vrathasha V, Alapati T, He J, et al. Dual SMAD inhibition and Wnt inhibition enable efficient and reproducible differentiations of induced pluripotent stem cells into retinal ganglion cells. Sci Rep. 2020;10(1):11828. 10.1038/s41598-020-68811-8. 32678240 10.1038/s41598-020-68811-8 PMC7366935

[43] Rabesandratana O, Chaffiol A, Mialot A, Slembrouck-Brec A, Joffrois C, Nanteau C, et al. Generation of a transplantable population of human iPSC-derived retinal ganglion cells. Front Cell Dev Biol. 2020;8:585675. 10.3389/fcell.2020.585675. 33195235 10.3389/fcell.2020.585675 PMC7652757

[44] Aparicio JG, Hopp H, Choi A, Mandayam Comar J, Liao VC, Harutyunyan N, et al. Temporal expression of CD184(CXCR4) and CD171(L1CAM) identifies distinct early developmental stages of human retinal ganglion cells in embryonic stem cell derived retina. Exp Eye Res. 2017;154:177–89. 10.1016/j.exer.2016.11.013. 27867005 10.1016/j.exer.2016.11.013 PMC5359064

[45] Gill KP, Hung SS, Sharov A, Lo CY, Needham K, Lidgerwood GE, et al. Enriched retinal ganglion cells derived from human embryonic stem cells. Sci Rep. 2016;6(1):30552. 10.1038/srep30552. 27506453 10.1038/srep30552 PMC4978994

[46] Rodriguez AR, de Sevilla Muller LP, Brecha NC. The RNA binding protein RBPMS is a selective marker of ganglion cells in the mammalian retina. J Comp Neurol. 2014;522(6):1411–43. 10.1002/cne.23521. 24318667 10.1002/cne.23521 PMC3959221

[47] Gudiseva HV, Vrathasha V, He J, Bungatavula D, O’Brien JM, Chavali VRM. Single cell sequencing of induced pluripotent stem cell derived retinal ganglion cells (iPSC-RGC) reveals distinct molecular signatures and RGC subtypes. Genes (Basel). 2021;12(12):2015. 10.3390/genes12122015. 34946963 10.3390/genes12122015 PMC8702079

[48] Langer KB, Ohlemacher SK, Phillips MJ, Fligor CM, Jiang P, Gamm DM, et al. Retinal ganglion cell diversity and subtype specification from human pluripotent stem cells. Stem Cell Reports. 2018;10(4):1282–93. 10.1016/j.stemcr.2018.02.010. 29576537 10.1016/j.stemcr.2018.02.010 PMC5998302

[49] Xiao D, Deng Q, Guo Y, Huang X, Zou M, Zhong J, et al. Generation of self-organized sensory ganglion organoids and retinal ganglion cells from fibroblasts. Sci Adv. 2020;6(22):eaaz5858. 10.1126/sciadv.aaz5858. 32523990 10.1126/sciadv.aaz5858 PMC7259937

[50] Nadal-Nicolas FM, Galindo-Romero C, Lucas-Ruiz F, Marsh-Amstrong N, Li W, Vidal-Sanz M, et al. Pan-retinal ganglion cell markers in mice, rats, and rhesus macaques. Zool Res. 2023;44(1):226–48. 10.24272/j.issn.2095-8137.2022.308. 36594396 10.24272/j.issn.2095-8137.2022.308 PMC9841181

[51] Artero Castro A, Rodriguez Jimenez FJ, Jendelova P, Erceg S. Deciphering retinal diseases through the generation of three dimensional stem cell-derived organoids: concise review. Stem Cells. 2019;37(12):1496–504. 10.1002/stem.3089. 31617949 10.1002/stem.3089 PMC6915910

[52] Garcia-Lopez M, Arenas J, Gallardo ME. Hereditary optic neuropathies: induced pluripotent stem cell-based 2D/3D approaches. Genes. 2021;12(1):112. 10.3390/genes12010112. 33477675 10.3390/genes12010112 PMC7831942

[53] Ji SL, Tang SB. Differentiation of retinal ganglion cells from induced pluripotent stem cells: a review. Int J Ophthalmol. 2019;12(1):152–60. 10.18240/ijo.2019.01.22. 30662854 10.18240/ijo.2019.01.22 PMC6326935

[54] Huang KC, Gomes C, Meyer JS. Retinal ganglion cells in a dish: current strategies and recommended best practices for effective in vitro modeling of development and disease. Handb Exp Pharmacol. 2023;281:83–102. 10.1007/164_2023_642. 36907969 10.1007/164_2023_642 PMC10497719

[55] Wong NK, Yip SP, Huang CL. Establishing functional retina in a dish: progress and promises of induced pluripotent stem cell-based retinal neuron differentiation. Int J Mol Sci. 2023;24(17):13652. 10.3390/ijms241713652. 37686457 10.3390/ijms241713652 PMC10487913

[56] Chambers SM, Fasano CA, Papapetrou EP, Tomishima M, Sadelain M, Studer L. Highly efficient neural conversion of human ES and iPS cells by dual inhibition of SMAD signaling. Nat Biotechnol. 2009;27(3):275–80. 10.1038/nbt.1529. 19252484 10.1038/nbt.1529 PMC2756723

[57] Elkabetz Y, Panagiotakos G, Al Shamy G, Socci ND, Tabar V, Studer L. Human ES cell-derived neural rosettes reveal a functionally distinct early neural stem cell stage. Genes Dev. 2008;22(2):152–65. 10.1101/gad.1616208. 18198334 10.1101/gad.1616208 PMC2192751

[58] Smith JR, Vallier L, Lupo G, Alexander M, Harris WA, Pedersen RA. Inhibition of Activin/Nodal signaling promotes specification of human embryonic stem cells into neuroectoderm. Dev Biol. 2008;313(1):107–17. 10.1016/j.ydbio.2007.10.003. 18022151 10.1016/j.ydbio.2007.10.003

[59] Lee J, Choi SH, Kim YB, Jun I, Sung JJ, Lee DR, et al. Defined conditions for differentiation of functional retinal ganglion cells from human pluripotent stem cells. Invest Ophthalmol Vis Sci. 2018;59(8):3531–42. 10.1167/iovs.17-23439. 30025074 10.1167/iovs.17-23439

[60] Vrathasha V, Nikonov S, Bell BA, He J, Bungatavula Y, Uyhazi KE, et al. Transplanted human induced pluripotent stem cells- derived retinal ganglion cells embed within mouse retinas and are electrophysiologically functional. iScience. 2022;25(11):105308. 10.1016/j.isci.2022.105308. 36388952 10.1016/j.isci.2022.105308 PMC9646916

[61] Gozlan S, Batoumeni V, Fournier T, Nanteau C, Potey A, Clémençon M, et al. Bankable human iPSC-derived retinal progenitors represent a valuable source of multipotent cells. Commun Biol. 2023. 10.1038/s42003-023-04956-2. 37479765 10.1038/s42003-023-04956-2 PMC10362027

[62] Eiraku M, Takata N, Ishibashi H, Kawada M, Sakakura E, Okuda S, et al. Self-organizing optic-cup morphogenesis in three-dimensional culture. Nature. 2011;472(7341):51–6. 10.1038/nature09941. 21475194 10.1038/nature09941

[63] Nakano T, Ando S, Takata N, Kawada M, Muguruma K, Sekiguchi K, et al. Self-formation of optic cups and storable stratified neural retina from human ESCs. Cell Stem Cell. 2012;10(6):771–85. 10.1016/j.stem.2012.05.009. 22704518 10.1016/j.stem.2012.05.009

[64] Chichagova V, Hilgen G, Ghareeb A, Georgiou M, Carter M, Sernagor E, et al. Human iPSC differentiation to retinal organoids in response to IGF1 and BMP4 activation is line- and method-dependent. Stem Cells. 2020;38(2):195–201. 10.1002/stem.3116. 31721366 10.1002/stem.3116 PMC7383896

[65] Kamarudin TA, Bojic S, Collin J, Yu M, Alharthi S, Buck H, et al. Differences in the activity of endogenous bone morphogenetic protein signaling impact on the ability of induced pluripotent stem cells to differentiate to corneal epithelial-like cells. Stem Cells. 2018;36(3):337–48. 10.1002/stem.2750. 29226476 10.1002/stem.2750 PMC5839253

[66] Li J, Chen Y, Ouyang S, Ma J, Sun H, Luo L, et al. Generation and staging of human retinal organoids based on Self-Formed ectodermal autonomous Multi-Zone system. Front Cell Dev Biol. 2021;9:732382. 10.3389/fcell.2021.732382. 34631711 10.3389/fcell.2021.732382 PMC8493070

[67] Livesey FJ, Cepko CL. Vertebrate neural cell-fate determination: lessons from the retina. Nat Rev Neurosci. 2001;2(2):109–18. 10.1038/35053522. 11252990 10.1038/35053522

[68] Finlay BL. The developing and evolving retina: using time to organize form. Brain Res. 2008;1192:5–16. 10.1016/j.brainres.2007.07.005. 17692298 10.1016/j.brainres.2007.07.005

[69] Kruczek K, Swaroop A. Pluripotent stem cell-derived retinal organoids for disease modeling and development of therapies. Stem Cells. 2020;38(10):1206–15. 10.1002/stem.3239. 32506758 10.1002/stem.3239 PMC7586922

[70] Daniszewski M, Senabouth A, Liang HH, Han X, Lidgerwood GE, Hernandez D, et al. Retinal ganglion cell-specific genetic regulation in primary open-angle glaucoma. Cell Genom. 2022;2(6):100142. 10.1016/j.xgen.2022.100142. 36778138 10.1016/j.xgen.2022.100142 PMC9903700

[71] Lee YJ, Jo DH. Retinal organoids from induced pluripotent stem cells of patients with inherited retinal diseases: a systematic review. Stem Cell Rev Rep. 2025;21(1):167–97. 10.1007/s12015-024-10802-7. 39422807 10.1007/s12015-024-10802-7 PMC11762450

[72] Shen Q, Goderie SK, Jin L, Karanth N, Sun Y, Abramova N, et al. Endothelial cells stimulate self-renewal and expand neurogenesis of neural stem cells. Science. 2004;304(5675):1338–40. 10.1126/science.1095505. 15060285 10.1126/science.1095505

[73] Inagaki S, Nakamura S, Kuse Y, Aoshima K, Funato M, Shimazawa M, et al. Establishment of vascularized human retinal organoids from induced pluripotent stem cells. Stem Cells. 2025;43(3). 10.1093/stmcls/sxae093. 10.1093/stmcls/sxae093 40037696

[74] Parkhurst CN, Yang G, Ninan I, Savas JN, Yates JR 3rd, Lafaille JJ, et al. Microglia promote learning-dependent synapse formation through brain-derived neurotrophic factor. Cell. 2013;155(7):1596–609. 10.1016/j.cell.2013.11.030. 24360280 10.1016/j.cell.2013.11.030 PMC4033691

[75] Paolicelli RC, Bolasco G, Pagani F, Maggi L, Scianni M, Panzanelli P, et al. Synaptic pruning by microglia is necessary for normal brain development. Science. 2011;333(6048):1456–8. 10.1126/science.1202529. 21778362 10.1126/science.1202529

[76] Lobov IB, Rao S, Carroll TJ, Vallance JE, Ito M, Ondr JK, et al. WNT7b mediates macrophage-induced programmed cell death in patterning of the vasculature. Nature. 2005;437(7057):417–21. 10.1038/nature03928. 16163358 10.1038/nature03928 PMC4259146

[77] Usui-Ouchi A, Giles S, Harkins-Perry S, Mills EA, Bonelli R, Wei G, et al. Integrating human iPSC-derived macrophage progenitors into retinal organoids to generate a mature retinal microglial niche. Glia. 2023;71(10):2372–82. 10.1002/glia.24428. 37335016 10.1002/glia.24428

[78] Schmied V, Korkut-Demirbas M, Venturino A, Maya-Arteaga JP, Siegert S. Microglia determine an immune-challenged environment and facilitate ibuprofen action in human retinal organoids. J Neuroinflammation. 2025;22(1):98. 10.1186/s12974-025-03366-x. 40181459 10.1186/s12974-025-03366-x PMC11966913

[79] Gao ML, Zhang X, Han F, Xu J, Yu SJ, Jin K, et al. Functional microglia derived from human pluripotent stem cells empower retinal organs. Sci China Life Sci. 2022;65(6):1057–71. 10.1007/s11427-021-2086-0. 35451725 10.1007/s11427-021-2086-0

[80] Capowski EE, Samimi K, Mayerl SJ, Phillips MJ, Pinilla I, Howden SE, et al. Reproducibility and staging of 3D human retinal organoids across multiple pluripotent stem cell lines. Development. 2019;146(1):dev171686. 10.1242/dev.171686. 30567931 10.1242/dev.171686 PMC6340149

[81] Wahlin KJ, Maruotti JA, Sripathi SR, Ball J, Angueyra JM, Kim C, et al. Photoreceptor outer segment-like structures in long-term 3D retinas from human pluripotent stem cells. Sci Rep. 2017;7(1):766. 10.1038/s41598-017-00774-9. 28396597 10.1038/s41598-017-00774-9 PMC5429674

[82] Murcia-Belmonte V, Erskine L. Wiring the binocular visual pathways. Int J Mol Sci. 2019. 10.3390/ijms20133282. 31277365 10.3390/ijms20133282 PMC6651880

[83] Fligor CM, Lavekar SS, Harkin J, Shields PK, VanderWall KB, Huang KC, et al. Extension of retinofugal projections in an assembled model of human pluripotent stem cell-derived organoids. Stem Cell Reports. 2021;16(9):2228–41. 10.1016/j.stemcr.2021.05.009. 34115986 10.1016/j.stemcr.2021.05.009 PMC8452489

[84] Luo Z, Chang KC, Wu S, Sun C, Xia X, Nahmou M, et al. Directly induced human retinal ganglion cells mimic fetal RGCs and are neuroprotective after transplantation in vivo. Stem Cell Reports. 2022;17(12):2690–703. 10.1016/j.stemcr.2022.10.011. 36368332 10.1016/j.stemcr.2022.10.011 PMC9768574

[85] Liou RH, Chen SW, Cheng HC, Wu PC, Chang YF, Wang AG, et al. The efficient induction of human retinal ganglion-like cells provides a platform for studying optic neuropathies. Cell Mol Life Sci. 2023;80(8):239. 10.1007/s00018-023-04890-w. 37540379 10.1007/s00018-023-04890-w PMC10403410

[86] Agarwal D, Dash N, Mazo KW, Chopra M, Avila MP, Patel A, et al. Human retinal ganglion cell neurons generated by synchronous BMP inhibition and transcription factor mediated reprogramming. NPJ Regen Med. 2023;8(1):55. 10.1038/s41536-023-00327-x. 37773257 10.1038/s41536-023-00327-x PMC10541876

[87] Orieux G, Rabesandratana O, Gagliardi G, Goureau O. Generation and isolation of retinal ganglion cells and photoreceptors from human iPSC-Derived retinal organoids by Magnetic-Activated cell sorting. Brain organoid research. Neuromethods: Springer US; 2023. pp. 67–80.

[88] Sutermaster BA, Darling EM. Considerations for high-yield, high-throughput cell enrichment: fluorescence versus magnetic sorting. Sci Rep. 2019;9(1):227. 10.1038/s41598-018-36698-1. 30659223 10.1038/s41598-018-36698-1 PMC6338736

[89] Bhattacharjee J, Das B, Mishra A, Sahay P, Upadhyay P. Monocytes isolated by positive and negative magnetic sorting techniques show different molecular characteristics and immunophenotypic behaviour. F1000Res. 2017;6:2045. 10.12688/f1000research.12802.3. 29636897 10.12688/f1000research.12802.1 PMC5871943

[90] Gao F, Li T, Hu J, Zhou X, Wu J, Wu Q. Comparative analysis of three purification protocols for retinal ganglion cells from rat. Mol Vis. 2016;22:387–400. 27122968 PMC4844924

[91] Daniszewski M, Senabouth A, Nguyen QH, Crombie DE, Lukowski SW, Kulkarni T, et al. Single cell RNA sequencing of stem cell-derived retinal ganglion cells. Sci Data. 2018;5(1):180013. 10.1038/sdata.2018.13. 29437159 10.1038/sdata.2018.13 PMC5810423

[92] Lei Q, Zhang R, Yuan F, Xiang M. Integration and differentiation of transplanted human iPSC-derived retinal ganglion cell precursors in murine retinas. Int J Mol Sci. 2024;25(23):12947. 10.3390/ijms252312947. 39684658 10.3390/ijms252312947 PMC11641514

[93] Edo A, Sugita S, Futatsugi Y, Sho J, Onishi A, Kiuchi Y, et al. Capacity of retinal ganglion cells derived from human induced pluripotent stem cells to suppress T-cells. Int J Mol Sci. 2020;21(21):7831. 10.3390/ijms21217831. 33105725 10.3390/ijms21217831 PMC7660053

[94] Hong S, Iizuka Y, Kim CY, Seong GJ. Isolation of primary mouse retinal ganglion cells using immunopanning-magnetic separation. Mol Vis. 2012;18:2922–30. 23233794 PMC3519380

[95] Zhang XM, Li Liu DT, Chiang SW, Choy KW, Pang CP, Lam DS, et al. Immunopanning purification and long-term culture of human retinal ganglion cells. Mol Vis. 2010;16:2867–72. 21203402 PMC3012647

[96] Koumas L, Smith TJ, Feldon S, Blumberg N, Phipps RP. Thy-1 expression in human fibroblast subsets defines myofibroblastic or lipofibroblastic phenotypes. Am J Pathol. 2003;163(4):1291–300. 10.1016/S0002-9440(10)63488-8. 14507638 10.1016/S0002-9440(10)63488-8 PMC1868289

[97] Hameed SS, Sharma TP. Generation of retinal ganglion cells from reprogrammed keratocytes of non-glaucoma and glaucoma donors. Curr Protoc. 2025;5(1):e70091. 10.1002/cpz1.70091. 39781605 10.1002/cpz1.70091 PMC11713219

[98] Yang YP, Chang YL, Lai YH, Tsai PH, Hsiao YJ, Nguyen LH, et al. Retinal circular RNA hsa_circ_0087207 expression promotes apoptotic cell death in induced pluripotent stem cell-derived leber’s hereditary optic neuropathy-like models. Biomedicines. 2022;10(4):788. 10.3390/biomedicines10040788. 35453537 10.3390/biomedicines10040788 PMC9027941

[99] VanderWall KB, Huang KC, Pan Y, Lavekar SS, Fligor CM, Allsop AR, et al. Retinal ganglion cells with a glaucoma OPTN(E50K) mutation exhibit neurodegenerative phenotypes when derived from three-dimensional retinal organoids. Stem Cell Reports. 2020;15(1):52–66. 10.1016/j.stemcr.2020.05.009. 32531194 10.1016/j.stemcr.2020.05.009 PMC7363877

[100] Chien Y, Yang YP, Lin TC, Chiou GY, Yarmishyn AA, Wang CH, et al. Reprogramming patient-induced pluripotent stem cell-specific retinal organoids for deciphering epigenetic modifications of RNA methylation. J Chin Med Assoc. 2025;88(2):116–25. 10.1097/JCMA.0000000000001198. 39710870 10.1097/JCMA.0000000000001198 PMC12718864

[101] Sucher NJ, Cheng TP, Lipton SA. Cryopreservation of postnatal rat retinal ganglion cells: persistence of voltage- and ligand-gated ionic currents. Neuroscience. 1991;43(1):135–50. 10.1016/0306-4522(91)90423-l. 1717883 10.1016/0306-4522(91)90423-l

[102] Papaioannou S, Medini P. Advantages, pitfalls, and developments of all optical interrogation strategies of microcircuits in vivo. Front Neurosci. 2022;16:859803. 10.3389/fnins.2022.859803. 35837124 10.3389/fnins.2022.859803 PMC9274136

[103] Tanaka T, Yokoi T, Tamalu F, Watanabe S, Nishina S, Azuma N. Generation of retinal ganglion cells with functional axons from human induced pluripotent stem cells. Sci Rep. 2015;5(1):8344. 10.1038/srep08344. 25666360 10.1038/srep08344 PMC4322369

[104] Yuste R, Katz LC. Control of postsynaptic Ca2 + influx in developing neocortex by excitatory and inhibitory neurotransmitters. Neuron. 1991;6(3):333–44. 10.1016/0896-6273(91)90243-s. 1672071 10.1016/0896-6273(91)90243-s

[105] Kerr JN, Greenberg D, Helmchen F. Imaging input and output of neocortical networks in vivo. Proc Natl Acad Sci U S A. 2005;102(39):14063–8. 10.1073/pnas.0506029102. 16157876 10.1073/pnas.0506029102 PMC1201343

[106] Zhang X, Tenerelli K, Wu S, Xia X, Yokota S, Sun C, et al. Cell transplantation of retinal ganglion cells derived from hESCs. Restor Neurol Neurosci. 2020;38(2):131–40. 10.3233/RNN-190941. 31815704 10.3233/RNN-190941

[107] Medini P. Cell-type-specific sub- and suprathreshold receptive fields of layer 4 and layer 2/3 pyramids in rat primary visual cortex. Neuroscience. 2011;190:112–26. 10.1016/j.neuroscience.2011.05.026. 21704132 10.1016/j.neuroscience.2011.05.026

[108] Chen M, Li Y, Yang M, Chen X, Chen Y, Yang F, et al. A new method for quantifying mitochondrial axonal transport. Protein Cell. 2016;7(11):804–19. 10.1007/s13238-016-0268-3. 27225265 10.1007/s13238-016-0268-3 PMC5084152

[109] Sleigh JN, Vagnoni A, Twelvetrees AE, Schiavo G. Methodological advances in imaging intravital axonal transport. F1000Res. 2017;6:200. 10.12688/f1000research.10433.1. 28344778 10.12688/f1000research.10433.1 PMC5333613

[110] Tian N. Developmental mechanisms that regulate retinal ganglion cell dendritic morphology. Dev Neurobiol. 2011;71(12):1297–309. 10.1002/dneu.20900. 21542137 10.1002/dneu.20900 PMC3923654

[111] VanderWall KB, Vij R, Ohlemacher SK, Sridhar A, Fligor CM, Feder EM, et al. Astrocytes regulate the development and maturation of retinal ganglion cells derived from human pluripotent stem cells. Stem Cell Reports. 2019;12(2):201–12. 10.1016/j.stemcr.2018.12.010. 30639213 10.1016/j.stemcr.2018.12.010 PMC6373493

[112] Cowan CS, Renner M, De Gennaro M, Gross-Scherf B, Goldblum D, Hou Y, et al. Cell types of the human retina and its organoids at Single-Cell resolution. Cell. 2020;182(6):1623–e4034. 10.1016/j.cell.2020.08.013. 32946783 10.1016/j.cell.2020.08.013 PMC7505495

[113] Hallam D, Hilgen G, Dorgau B, Zhu L, Yu M, Bojic S, et al. Human-induced pluripotent stem cells generate light responsive retinal organoids with variable and nutrient-dependent efficiency. Stem Cells. 2018;36(10):1535–51. 10.1002/stem.2883. 30004612 10.1002/stem.2883 PMC6392112

[114] Celiker C, Weissova K, Cerna KA, Oppelt J, Dorgau B, Gambin FM, et al. Light-responsive microRNA molecules in human retinal organoids are differentially regulated by distinct wavelengths of light. iScience. 2023;26(7):107237. 10.1016/j.isci.2023.107237. 37485345 10.1016/j.isci.2023.107237 PMC10362355

[115] Li J, Wang J, Ibarra IL, Cheng X, Luecken MD, Lu J, et al. Integrated multi-omics single cell atlas of the human retina. Res Sq. 2023:rs. 3. rs-3471275. 10.21203/rs.3.rs-3471275/v1.

[116] Yan W, Peng YR, van Zyl T, Regev A, Shekhar K, Juric D, et al. Cell atlas of the human fovea and peripheral retina. Sci Rep. 2020a;10(1):9802. 10.1038/s41598-020-66092-9. 32555229 10.1038/s41598-020-66092-9 PMC7299956

[117] Lukowski SW, Lo CY, Sharov AA, Nguyen Q, Fang L, Hung SS, et al. A single-cell transcriptome atlas of the adult human retina. EMBO J. 2019;38(18):e100811. 10.15252/embj.2018100811. 31436334 10.15252/embj.2018100811 PMC6745503

[118] Wang SK, Nair S, Li R, Kraft K, Pampari A, Patel A, et al. Single-cell multiome of the human retina and deep learning nominate causal variants in complex eye diseases. Cell Genomics. 2022;2(8):100164. 10.1016/j.xgen.2022.100164. 36277849 10.1016/j.xgen.2022.100164 PMC9584034

[119] Wahle P, Brancati G, Harmel C, He Z, Gut G, Del Castillo JS, et al. Multimodal spatiotemporal phenotyping of human retinal organoid development. Nat Biotechnol. 2023;41(12):1765–75. 10.1038/s41587-023-01747-2. 37156914 10.1038/s41587-023-01747-2 PMC10713453

[120] Morgan JE, Bevan RJ, Cimaglia G. Retinal Ganglion Cell Subtypes and Their Vulnerability in Glaucoma. In: *Glaucoma: methods and protocols*. Springer US; 2025. p. 191–205. 10.1007/978-1-0716-4140-8_16 39433677

[121] Grunert U, Martin PR. Cell types and cell circuits in human and non-human primate retina. Prog Retin Eye Res. 2020;100844. 10.1016/j.preteyeres.2020.100844. 10.1016/j.preteyeres.2020.100844 32032773

[122] Dacey DM, Petersen MR. Dendritic field size and morphology of midget and parasol ganglion cells of the human retina. Proc Natl Acad Sci U S A. 1992;89(20):9666–70. 10.1073/pnas.89.20.9666. 1409680 10.1073/pnas.89.20.9666 PMC50193

[123] Yamasaki EN, Ramoa AS. Dendritic remodelling of retinal ganglion cells during development of the rat. J Comp Neurol. 1993;329(2):277–89. 10.1002/cne.903290209. 8454733 10.1002/cne.903290209

[124] Reinhard K, Münch TA. Visual properties of human retinal ganglion cells. PLoS One. 2021;16(2):e0246952. 10.1371/journal.pone.0246952. 33592045 10.1371/journal.pone.0246952 PMC7886124

[125] Kaplan E, Shapley RM. The primate retina contains two types of ganglion cells, with high and low contrast sensitivity. Proc Natl Acad Sci. 1986;83(8):2755–7. 10.1073/pnas.83.8.2755. 3458235 10.1073/pnas.83.8.2755 PMC323379

[126] Crook JD, Davenport CM, Peterson BB, Packer OS, Detwiler PB, Dacey DM. Parallel ON and OFF cone bipolar inputs establish spatially coextensive receptive field structure of blue-yellow ganglion cells in primate retina. J Neurosci. 2009;29(26):8372–87. 10.1523/jneurosci.1218-09.2009. 19571128 10.1523/JNEUROSCI.1218-09.2009 PMC2733872

[127] Schmidt TM, Kofuji P. Functional and morphological differences among intrinsically photosensitive retinal ganglion cells. J Neurosci. 2009;29(2):476–82. 10.1523/jneurosci.4117-08.2009. 19144848 10.1523/JNEUROSCI.4117-08.2009 PMC2752349

[128] Huang W, Xu Q, Su J, Tang L, Hao Z-Z, Xu C, et al. Linking transcriptomes with morphological and functional phenotypes in mammalian retinal ganglion cells. Cell Rep. 2022;40(11):111322. 10.1016/j.celrep.2022.111322. 36103830 10.1016/j.celrep.2022.111322

[129] Wang K, Gaitsch H, Poon H, Cox NJ, Rzhetsky A. Classification of common human diseases derived from shared genetic and environmental determinants. Nat Genet. 2017;49(9):1319–25. 10.1038/ng.3931. 28783162 10.1038/ng.3931 PMC5577363

[130] Han X, Gharahkhani P, Hamel AR, Ong JS, Renteria ME, Mehta P, et al. Large-scale multitrait genome-wide association analyses identify hundreds of glaucoma risk loci. Nat Genet. 2023;55(7):1116–25. 10.1038/s41588-023-01428-5. 37386247 10.1038/s41588-023-01428-5 PMC10335935

[131] Rezaie T, Child A, Hitchings R, Brice G, Miller L, Coca-Prados M, et al. Adult-onset primary open-angle glaucoma caused by mutations in optineurin. Science. 2002;295(5557):1077–9. 10.1126/science.1066901. 11834836 10.1126/science.1066901

[132] De Marco N, Buono M, Troise F, Diez-Roux G. Optineurin increases cell survival and translocates to the nucleus in a Rab8-dependent manner upon an apoptotic stimulus. J Biol Chem. 2006;281(23):16147–56. 10.1074/jbc.M601467200. 16569640 10.1074/jbc.M601467200

[133] Sahlender DA, Roberts RC, Arden SD, Spudich G, Taylor MJ, Luzio JP, et al. Optineurin links myosin VI to the Golgi complex and is involved in Golgi organization and exocytosis. J Cell Biol. 2005;169(2):285–95. 10.1083/jcb.200501162. 15837803 10.1083/jcb.200501162 PMC2171882

[134] Gomes C, VanderWall KB, Pan Y, Lu X, Lavekar SS, Huang KC, et al. Astrocytes modulate neurodegenerative phenotypes associated with glaucoma in OPTN(E50K) human stem cell-derived retinal ganglion cells. Stem Cell Reports. 2022;17(7):1636–49. 10.1016/j.stemcr.2022.05.006. 35714595 10.1016/j.stemcr.2022.05.006 PMC9287669

[135] Huang KC, Gomes C, Shiga Y, Belforte N, VanderWall KB, Lavekar SS, et al. Acquisition of neurodegenerative features in isogenic OPTN(E50K) human stem cell-derived retinal ganglion cells associated with autophagy disruption and mTORC1 signaling reduction. Acta Neuropathol Commun. 2024;12(1):164. 10.1186/s40478-024-01872-2. 39425218 10.1186/s40478-024-01872-2 PMC11487784

[136] Wong RCB, Lim SY, Hung SSC, Jackson S, Khan S, Van Bergen NJ, et al. Mitochondrial replacement in an iPSC model of Leber’s hereditary optic neuropathy. Aging. 2017;9(4):1341–50. 10.18632/aging.101231. 28455970 10.18632/aging.101231 PMC5425131

[137] Yu-Wai-Man P, Griffiths PG, Chinnery PF. Mitochondrial optic neuropathies - disease mechanisms and therapeutic strategies. Prog Retin Eye Res. 2011;30(2):81–114. 21112411 10.1016/j.preteyeres.2010.11.002 PMC3081075

[138] Zhang J, Liu X, Liang X, Lu Y, Zhu L, Fu R, et al. A novel ADOA-associated OPA1 mutation alters the mitochondrial function, membrane potential, ROS production and apoptosis. Sci Rep. 2017;7(1):5704. 10.1038/s41598-017-05571-y. 28720802 10.1038/s41598-017-05571-y PMC5515948

[139] Sladen PE, Jovanovic K, Guarascio R, Ottaviani D, Salsbury G, Novoselova T, et al. Modelling autosomal dominant optic atrophy associated with OPA1 variants in iPSC-derived retinal ganglion cells. Hum Mol Genet. 2022;31(20):3478–93. 10.1093/hmg/ddac128. 35652445 10.1093/hmg/ddac128 PMC9558835

[140] Lei Q, Xiang K, Cheng L, Xiang M. Human retinal organoids with an OPA1 mutation are defective in retinal ganglion cell differentiation and function. Stem Cell Reports. 2024;19(1):68-83. 10.1016/j.stemcr.2023.11.004. 10.1016/j.stemcr.2023.11.004 PMC10828684 38101398

[141] Doucette LP, Rasnitsyn A, Seifi M, Walter MA. The interactions of genes, age, and environment in glaucoma pathogenesis. Surv Ophthalmol. 2015;60(4):310–26. 10.1016/j.survophthal.2015.01.004. 25907525 10.1016/j.survophthal.2015.01.004

[142] Yang T-H, Kang EY-C, Lin P-H, Yu BB-C, Wang JH-H, Chen V, et al. Mitochondria in retinal ganglion cells: unraveling the metabolic nexus and oxidative stress. Int J Mol Sci. 2024b;25(16):8626. 10.3390/ijms25168626 10.3390/ijms25168626 PMC11354650 39201313

[143] Zibold J, Von Livonius B, Kolarova H, Rudolph G, Priglinger CS, Klopstock T, et al. Vitamin B12 in Leber hereditary optic neuropathy mutation carriers: a prospective cohort study. Orphanet J Rare Dis. 2022. 10.1186/s13023-022-02453-z. 35945620 10.1186/s13023-022-02453-z PMC9361590

[144] Kirkman MA, Yu-Wai-Man P, Korsten A, Leonhardt M, Dimitriadis K, De Coo IF, et al. Gene-environment interactions in Leber hereditary optic neuropathy. Brain. 2009;132(Pt 9):2317–26. 10.1093/brain/awp158. 19525327 10.1093/brain/awp158 PMC2732267

[145] Erchova I, Sun S, Votruba M. A perspective on accelerated aging caused by the genetic deficiency of the metabolic protein, OPA1. Front Neurol. 2021. 10.3389/fneur.2021.641259. 33927681 10.3389/fneur.2021.641259 PMC8076550

[146] Dorgau B, Georgiou M, Chaudhary A, Moya-Molina M, Collin J, Queen R, et al. Human retinal organoids provide a suitable tool for toxicological investigations: a comprehensive validation using drugs and compounds affecting the retina. Stem Cells Transl Med. 2022;11(2):159–77. 10.1093/stcltm/szab010. 35298655 10.1093/stcltm/szab010 PMC8929478

[147] Zhou X, Rui Y, Peng J, Wang Y, He Y, Wang C, et al. Transplantation of reprogrammed peripheral blood cells differentiates into retinal ganglion cells in the mouse eye with NMDA-induced injury. J Cell Physiol. 2021;236(12):8099–109. 10.1002/jcp.30464. 34101182 10.1002/jcp.30464

[148] Doss MX, Sachinidis A. Current challenges of iPSC-based disease modeling and therapeutic implications. Cells. 2019;8(5):403. 10.3390/cells8050403. 31052294 10.3390/cells8050403 PMC6562607

[149] Oswald J, Kegeles E, Minelli T, Volchkov P, Baranov P. Transplantation of miPSC/mESC-derived retinal ganglion cells into healthy and glaucomatous retinas. Mol Ther Methods Clin Dev. 2021;21:180–98. 10.1016/j.omtm.2021.03.004. 33816648 10.1016/j.omtm.2021.03.004 PMC7994731

[150] Wu S, Chang KC, Nahmou M, Goldberg JL. Induced pluripotent stem cells promote retinal ganglion cell survival after transplant. Invest Ophthalmol Vis Sci. 2018;59(3):1571–6. 10.1167/iovs.17-23648. 29625481 10.1167/iovs.17-23648 PMC5863687

[151] Boyd AS, Higashi Y, Wood KJ. Transplanting stem cells: potential targets for immune attack. Modulating the immune response against embryonic stem cell transplantation. Adv Drug Deliv Rev. 2005;57(13):1944–69. 10.1016/j.addr.2005.08.004. 16289432 10.1016/j.addr.2005.08.004

[152] Rabesandratana O, Goureau O, Orieux G. Pluripotent stem cell-based approaches to explore and treat optic neuropathies. Front Neurosci. 2018;12:651. 10.3389/fnins.2018.00651. 30294255 10.3389/fnins.2018.00651 PMC6158340

